# Toll-like receptor 4 pathway evolutionary trajectory and functional emergence

**DOI:** 10.3389/fimmu.2024.1494017

**Published:** 2025-01-20

**Authors:** Shailya Verma, Ramanathan Sowdhamini

**Affiliations:** ^1^ National Centre for Biological Sciences (TIFR), Bangalore, India; ^2^ Molecular Biophysics Unit, Indian Institute of Science, Bangalore, India; ^3^ Institute of Bioinformatics and Applied Biotechnology, Bangalore, India

**Keywords:** TLR adaptor proteins, TRAM, TRIF, orthologs, protein modeling, molecular dynamics simulations, residue network analysis, normal mode analysis

## Abstract

**Introduction:**

Toll-like receptors 4 (TLR4) recognize lipopolysaccharides (LPS) from bacteria as their conventional ligands and undergo downstream signaling to produce cytokines. They mediate the signaling either by the TIRAP-MyD88 complex or by the TRAM-TRIF complex. The MyD88 pathway is common to all other TLRs, whereas the TRAM-TRIF complex is largely exclusive to TLR4. Here we study the TIR domain of TRAM and TRIF ortholog proteins that are crucial for downstream signaling. Our previous work on pan-genome-wide survey, indicates *Callorhincus milli* to be the ancestral organism with both TRAM and TRIF proteins.

**Methods:**

To gain a deeper insight into the protein function and to compare them with *Homo sapiens* adaptor proteins, we modeled the docking of the TRAM–TRIF complex of representative organisms across various taxa. These modeling experiments provide insights to ascertain a possible interaction surface and calculate the energetics and electrostatic potential of the complex. Furthermore, this enables us to employ normal mode analysis (NMA) to examine fluctuating, interacting, and other specific residue clusters that could have a role in protein functioning in both *C. milli* and *H. sapiens*. We also performed molecular dynamics simulations of these complexes and cross-validated the functionally important residues using network parameters.

**Results:**

We compared the stoichiometry of TRAM–TRIF complexes and found that the tetrameric models (TRAM and TRIF dimer) were more stable than the trimeric model (TRAM dimer and TRIF monomer). While the critical residues of TIRAP, TRIF, and MyD88 were preserved, we also found that the important residues of TRAM signaling were not conserved in *C. milli*.

**Discussion:**

This suggests the presence of functional TIRAP–MyD88-mediated TLR4 signaling and TRIF-mediated TLR3 signaling in the ancestral species. The overall biological function of this signaling domain appears to be gradually acquired through the orchestration of several motifs through an evolutionary scale.

## 1 Introduction

Toll-like receptors (TLRs) are key pattern recognition receptors (PRRs) responsible for the identification of various pathogen-associated molecular patterns (PAMPs) and danger-associated molecular patterns (DAMPs). They are part of the mammalian innate immune system and provide protection from various pathogens. The TLRs are localized either on the plasma membrane or on endosomes. They have an extracellular domain (ECD) that recognizes or interacts with the ligand, a transmembrane domain (TMD), and an intracellular Toll/interleukin 1 receptor (TIR) domain. One of the TLRs, TLR4, identifies lipopolysaccharide (LPS) as its primary ligand. The interaction between TLR4 and LPS results in two routes of downstream signaling, which are mediated by four adaptor proteins.

The conventional route is mediated by the interaction of TIR adaptor protein (TIRAP) and myeloid differentiation primary response protein 88 (MyD88). This is common for all TLRs localized to the plasma membrane. Additionally, TLR4 and TLR2 also mediate signaling by the TIR domain-containing adapter molecule 2 (TICAM2/TRAM) and TIR domain-containing adapter molecule 1 (TICAM1/TRIF) to produce type I interferons (IFNs) (1, 2). This signaling pathway is mediated by the accessory proteins cluster of differentiation 14 (CD14) and myeloid differentiation factor-2 (MD2). The CD14 protein is localized to the plasma membrane, where it binds to LPS and recruits it to TLR4. Furthermore, TLR4 recognizes LPS with help from MD2 protein and undergoes dimerization in the process. The MyD88 pathway and TRAM-mediated pathway are competitive and mutually exclusive to each other and the latter gets activated when the complex internalizes into endosomes (3).

TLRs are essential proteins that connect innate and adaptive immunity by recognizing ligands, and their signaling leads to the production of cytokines (4). Several studies have been conducted on the traditional MyD88–TIRAP pathway in an attempt to comprehend the evolution of these signaling pathways (5, 6); however, the TRAM–TRIF pathway has not been fully explored. Recently, we had reported the evolution of TRAM and TRIF proteins across various taxa from the tree of life (7). The conservation pattern of several key residues and motif patterns among orthologs suggest a potential pathway in non-mammals. These conserved residues are the AEDD site (A85, E86, D87, and D88), which is important for the upstream interaction of the TIR domains of TRAM and TLR4; BB loop residues, which are crucial for TRAM dimer formation; the TS site (T155 and S156), important for the downstream interaction between the TIR domains of TRAM and TRIF; and the Y167 phosphorylation site (8, 9).

**Figure 1:**
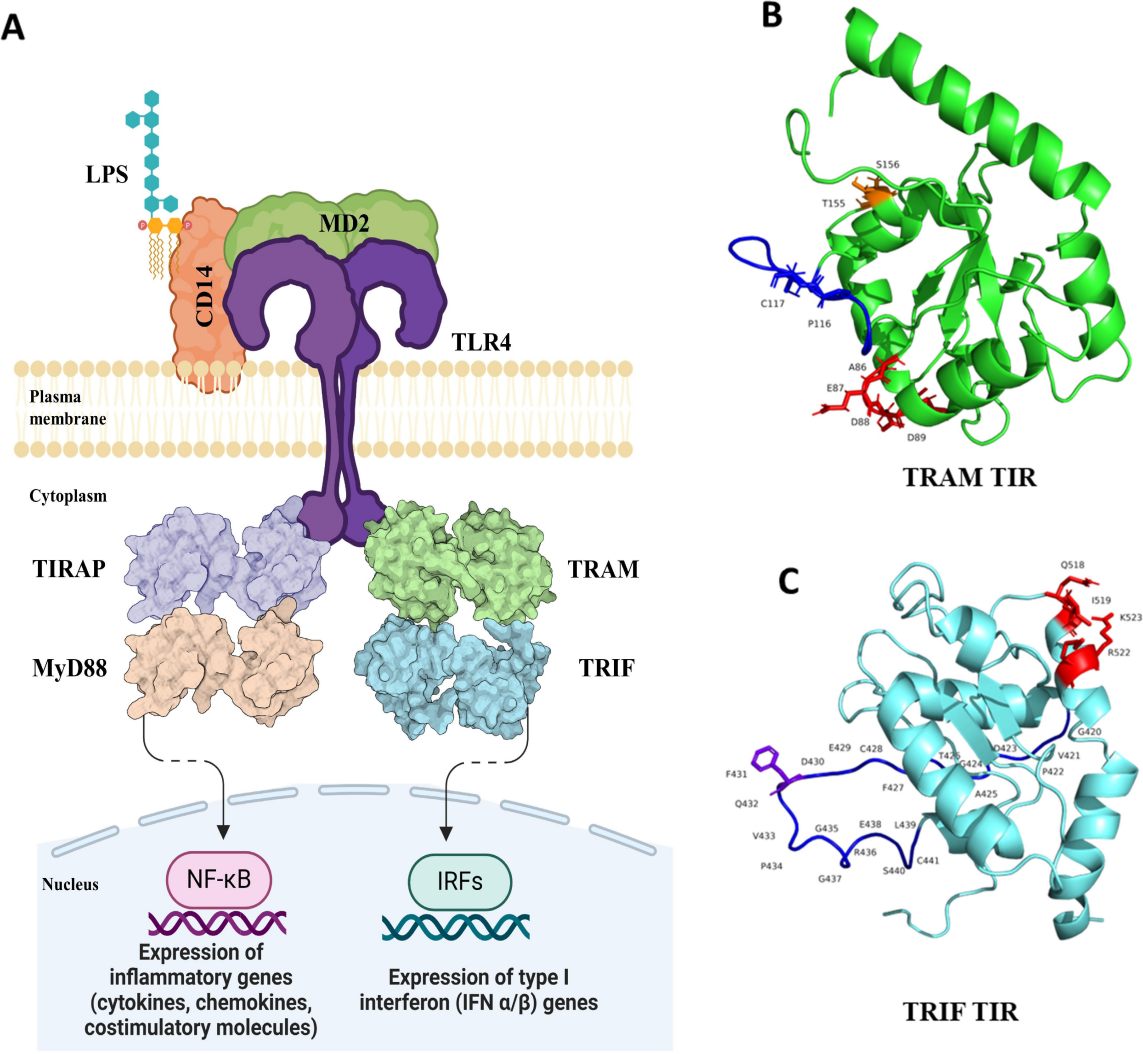
**(A)** Schematic of Toll-like receptor 4 signaling with the adaptor molecules, TIRAP, MyD88, and TRAM, TRIF-mediated pathways. The dashed arrow represents multiple other mediators in between that ultimately leads to IRF (IRF3 and IRF7) production and expression of type I interferon (IFN α/β) genes. Image created using BioRender. **(B)** Structure of the TRAM–TIR domain with highlighted key residues (AEDD site: red, BB loop: blue, P116 and C117: blue, TS site: orange). **(C)** Structure of TRIF–TIR with highlighted key residues (QI and RK site: red, BB loop: blue, F431: purple).

Mutations at two residues in the BB loop, P116 and C117, lead to the abrogation of downstream signaling (10, 11). Human TRAM has a myristoylation motif that is responsible for the localization of the protein to the plasma membrane (12). Moreover, the TRAF 6 binding motif on TRAM ensures the activation of response by TLR4. The schematic of the TLR-4 pathway with key residues is shown in **Figure 1**. On the other hand, human TRIF protein has three domains, the N-terminal domain (NTD), TIR domain, and RIP homotypic interaction motif (RHIM) domain. The NTD domain is responsible for the production of nuclear factor kappa B (NF-κB) and interferon regulatory factor 3 (IRF3). The RHIM domain is responsible for the TRIF-induced apoptosis (13). TRIF harbors the pLxIS motif that contains the phosphorylation site used for IRF3 activation; it also contains the TRAF6 binding motif that activates NF-κB production and the RHIM motif in the RHIM domain (14). TRIF–TIR has QI (Q518 and I519) and RK (R522 and K523) sites, which are important for the interaction with TRAM–TIR. Additionally, F431 is identified as a crucial residue that facilitates the interaction between the TRIF–TIR domain and its TRIF–NTD, thereby maintaining an autoinhibitory state (8, 15).

We considered the most crucial residues of human TRIF and TRAM and studied the conservation of the key residues in the functional TIR domain of both proteins, which are significant for dimer formation and downstream signaling (8). These TIR domains provide a scaffold for protein interactions and help in recruiting downstream effectors, such as IRAK, TRAF6, and TBK (16). Even though these TIR domains are common to different TLRs, interleukin receptors, and adaptor proteins, they have sequence divergence and functional specificity (17). We have modeled these TRAM–TRIF complexes assuming both trimeric and tetrameric orientations. We further used the best model to hypothesize the interaction pattern in case of representative organisms across various taxa. We have also used normal mode analysis (NMA) and molecular dynamics (MD) simulation to understand the complexes and to examine if the primitive orthologs of this complex have functional capability for signal transduction.

## 2 Materials and methods

### 2.1 Homologous protein analysis

The ortholog hits were selected based on specific and conserved positions of amino acid for the TIR_2 domain of TRAM (PDB ID: 2M1W) and TRIF (PDB ID: 2M1X) (7). These ortholog sequences were searched using 30 mammalian query sequences across a non-redundant database by genome-wide search method using CS-BLAST methods. These hits were filtered based on query coverage cutoff (>50%) and percentage identity cutoff (>30%). The sequences were also categorized based on conserved motifs and domain patterns. The detailed study of these sequences is presented in one of the previous studies from our lab (7). CONSURF2016 was used on these sequence sets, to visualize conservation in protein sequence among evolutionary orthologs with default settings using the multiple sequence alignment of TRAM and TRIF orthologs. These mappings were done with respect to the TIR_2 domain on PDB structure (ID: 2M1W and ID:2M1X), and in case of mutations (2M1W: H117C, 2M1X: P434H) they were reverted to wild type (18). These sequences were further analyzed using the evolutionary trace (ET) method to compute the relative rank of functional and structural position among protein homologs with default parameters using the protein sequence in fasta format (19). Furthermore, visualCMAT was used to analyze coevolving residues (20). The web server was used to find the coevolving residues using multiple aligned sequence data and protein TIR domain structure with default parameters.

### 2.2 Protein stability analysis

The coevolving residue pairs that belong to the highly conserved category from CONSURF analysis (score > 6) were further analyzed. These pairs were first checked for the frequency (>1%) of their occurrences in the orthologs. The corresponding amino acid changes were then incorporated using FoldX 5.0 (21). The RepairPDB command was used initially on TRIF–TIR and TRAM–TIR domain structures (2M1W; H117C, 2M1X; P434H) to identify residues with bad torsion angles, van der Waals clashes, or total energy and repairs them. The other parameters include pH = 7, temperature = 298 K, ionStrength = 0.05M, and vdwDesign = 2. After repairing the structures, the BuildModel command was used to mutate the residues from coevolving pairs to differently observed combinations. These runs were iterated five times with the same parameters as in RepairPDB and then the Average score for ΔΔ*G* kcal/mol (Δ*G*_mut_ − Δ*G*_wt_) was calculated. The results were binned in different categories based on the ΔΔ*G* values as follows: highly stabilizing (ΔΔ*G* < −1.84 kcal/mol), stabilizing (−1.84 kcal/mol ≤ ΔΔ*G* < −0.92 kcal/mol), slightly stabilizing (−0.92 kcal/mol ≤ ΔΔ*G* < −0.46 kcal/mol), neutral (−0.46 kcal/mol < ΔΔ*G* ≤ +0.46 kcal/mol), slightly destabilizing (+0.46 kcal/mol < ΔΔ*G* ≤ +0.92 kcal/mol), destabilizing (+0.92 kcal/mol < ΔΔ*G* ≤ +1.84 kcal/mol), and highly destabilizing (ΔΔ*G* > +1.84 kcal/mol).

### 2.3 Protein modeling and docking of trimer complex

Multiple approaches were used for modeling proteins, which were dimer, trimer, and tetramer complexes. The dimer model of TRAM protein had been established using various parameters, in a previous study from our lab (22). In one of the approaches, the TRAM MD stabilized structure was used as a starting dimer model, and using the protein docking algorithm, the TRAM dimer and TRIF complex was modeled. The HADDOCK 2.4 and HDOCK webservers were used to dock the models by both blind and guided docking (23–25). In HADDOCK 2.4, we have used individual modeled TIR protein and specified the protein–protein interaction option with other default parameters. In case of guided docking, the residue positions of the AEDD and TS sites from TRAM and QI and RK sites of TRIF were defined for the process, as per the evidence from the literature (8). While using HDOCK, both methods of docking were tried by using the modeled protein directly or by using protein sequence to model the protein using its internal algorithm and then performing docking.

Following the current trend in the field, we also used the AlphaFold Colab notebook that is based on a slightly modified version of AlphaFold v2.3.2 (https://colab.research.google.com/github/deepmind/alphafold/blob/main/notebooks/AlphaFold.ipynb#scrollTo=rowN0bVYLe9n). This is a template independent model, which is trained on the BFD database (26).

Six different approaches were used to model the TRAM–TRIF TRIMER complex as reported in previous literature (8). In approach 1, we used the AlphaFold modeled TIR of TRAM and TRIF proteins and docked them using the known interacting residues from literature (AEDD and TS sites of TRAM and QI and RK sites of TRIF) using HADDOCK. In approach 2, we used the MD stabilized final frame structure (200th ns frame) of TRAM dimer and performed blind docking using HDOCK. In approach 3, we used structural modeling of TRAM and TRIF from their respective sequence followed by blind docking using HDOCK. In approach 4, we completely used the AlphaFold to build the multimer complex. Approaches 5 and 6 were somewhat similar to approach 4. Here, we used the MD stabilized final frame structure of TRAM dimer and did guided docking [AEDD and TS sites of TRAM and QI and RK sites of TRIF (8)] using HADDOCK and HDOCK methods, respectively.

Later, these complexes were analyzed based on the positioning of the key residues, positioning of the BB loop, and energy calculations at the interface [calculated using PPCheck (27)]. We found substantial evidence from the literature that the HDOCK is more efficient as compared to other methods, and based on validation parameters, it stands as the best method (28). Thereby, we used the 6th approach model of the TRAM–TRIF trimer complex for further analysis. The structure of the trimeric complex and its energies is shown in **Supplementary Figure S10**.

### 2.4 Protein modeling and docking of the tetramer complex

There is limited literature evidence of the TRIF dimer interacting with the TRAM dimer (29, 30). To investigate this possibility further, we modeled the TRIF dimer using multiple approaches. We used HDOCK for blind and guided docking of the TRIF dimer (dimer along the BB loop, as observed in most TIR domain interactions). In the other approach, our most stabilized structure of the TRAM dimer was used as a template and homology modeling (HM) was performed for the TRIF dimer. We also used template-based TRIF dimer modeling using the available structure in PDB [TLR6 (PDB ID: 4OM7), TLR10 (PDB ID: 2J67), and IL-1RAPL (PDB ID: 1T3G)].

Furthermore, these six TRIF dimer models were used to establish the TRAM and TRIF tetramer complex. All these combinations were further compared based on the energetic values and significant residue positions. The electrostatics at the dimeric interface were also examined using the APBS plugin of the PyMOL software (31).

We have uploaded the Trimer and Tetramer model of *Homo sapiens* in Modelarchive. The other organisms are modeled by HM using the *H. sapiens* template.

The IDs of the uploaded models are as follows:

- ma-cxvbo, Human TRAM dimer and TRIF dimer (TETRAMER complex)
- ma-q7jrq, Human TRAM dimer and TRIF monomer (TRIMER complex)

### 2.5 Selection of representative organism

HM was performed for some of the representative organisms from different taxa across the tree of life using Modeller (32). In a previous study, we have traced the evolution of TRAM and TRIF protein from the oldest ancestors (7). We had analyzed the domain architecture and gained deep insights into the residue conservation pattern. From the corresponding study, the representative organisms were selected from different taxa, such that it consists of well-annotated TRAM–TIR and TRIF–TIR domains as it is crucial for signaling. *Callorhincus milli* (Chondrichthyes) was the oldest ancestor with both TRAM–TIR and TRIF–TIR domains (7). *Xenopus laevis* (Amphibians), *Chelonia mydas* (Cryptodira), *Crocodylus porosus* (Crocodylia), *Gekko japonicus* (Bifurcata), *Neopelma chrysocephalum* (Aves), and *Ornithorhynchus anatinus* (Mammalia) were chosen as representatives of each taxon. The NCBI IDs of the TRIF protein for the representative organisms and ruminants are as follows: *C. milli* (XP 007899298.1), *X. laevis* (XP 018109628.1), *C. mydas* (XP 007072279.1), *C. porosus* (XP 019406074.1), *G. japonicus* (XP 015276078.1), *N. chrysocephalum* (XP 027562012.1)*, O. anatinus* (XP 028906958.1), *H. sapiens* (NP 891549.1), *O. aries* (XP 004023546.3), *C. hircus* (XP 013820713.2), *Bubalus bubalis* (XP 006067185.2), *Bos mutus* (XP 014338163.1), *Bos indicus* x *Bos taurus* (XP 027403126.1), *Bos indicus* (XP 019819974.1), *Bos taurus* (AAI51623.1), *Bison bison bison* (XP 010826467.1), and *Odocoileus virginianus texanus* (XP 020769932.1).

The NCBS IDs of the TRAM protein for the representative organisms and ruminants are follows: *C. milli* (NP 001279313.1), *X. laevis* (XP 018119788.1), *C. mydas* (XP 007055469.2), *C. porosus* (XP 019410568.1), *G. japonicus* (XP 015271788.1), *N. chrysocephalum* (XP 027564076.1)*, O. anatinus* (NP 001191386.1), *H. sapiens* (NP 067681.1), *O. aries* (XP 004010232.2), *C. hircus* (AFN27530.1), *B. bubalis* (XP 006047327.1), *B. mutus* (XP 005902009.1), *B. taurus* (NP 001039921.1), *B. bison bison* (XP 010860382.1), and *O. virginianus texanus* (XP 020748203.1).

### 2.6 Normal mode analysis

The Bio3d package in R was used for comparative analysis of protein structures (33). NMA was performed to capture the large-scale molecular motions of the proteins. To obtain a detailed understanding at the all-atom level, an all-atom normal mode analysis (ENM) was also conducted (34). Hessian matrix was calculated for the protein complex and the lowest frequency modes were observed (35). The initial six modes represent the trivial modes with zero frequency corresponding to the rigid-body rotation and translation. The modeled protein complex was used as in input with the default parameters for the runs (http://thegrantlab.org/bio3d/reference/nma.html). We performed dynamic cross-correlation analysis, plotted the residue interaction network (RIN), and finally the network analysis (using the cna function). Several numbers of network communities were observed in each complex. Later, fluctuation and deformation analyses were performed as well. These measures provide us the amplitude of absolute atomic motions and the amount of local flexibility in the protein structure, respectively.

### 2.7 Molecular dynamics simulations

The complexes were also subjected to MD simulations to obtain a detailed understanding of the all-atom movements. Initially, the protein structure was prepared, using the protein preparation wizard of Maestro from the Schrodinger suite (36). The pre-processing was performed to cap the termini and by filling the missing residues, if any. Then, H-bond assignments were optimized, water molecules were deleted from the complex, and the overall structure was minimized. The system builder option was then used to prepare the system. The TIP4P explicit solvent model was chosen, and an orthorhombic box was defined with a minimum volume size (37). The OPLS4 force field was selected and the whole system was neutralized by adding an equivalent number of ions (38). Additionally, to mimic the physiological conditions, the salt concentration was maintained with 150 mM NaCl. The complex was further relaxed and a simulation of 200 ns was run at NPT conditions using Desmond from the Schrodinger suite (39). The trajectory was further converted and used for dynamic cross-correlation analysis using the Bio3d package (33).

### 2.8 Residue network analysis

The calculated MD trajectory was further used to perform dynamic residue network (DRN) analysis using multiple trajectory frames. For the above-mentioned analysis, a residue network graph was constructed using C-alpha atoms as nodes that were connected to each other by edges with a defined cutoff distance of 6.7 Å for each protein residue pair. Several DRN metrics like Betweenness Centrality (BC), Average Shortest Path Length (L), Closeness Centrality (CC), Eccentricity (ECC), Degree Centrality (DC), Eigencentrality (EC), Katz centrality (KC), and PageRank (PR) were calculated using the MDM-TASK-web platform (40).

## 3 Results

### 3.1 Residue conservation in TRAM and TRIF orthologs

**Figure 2:**
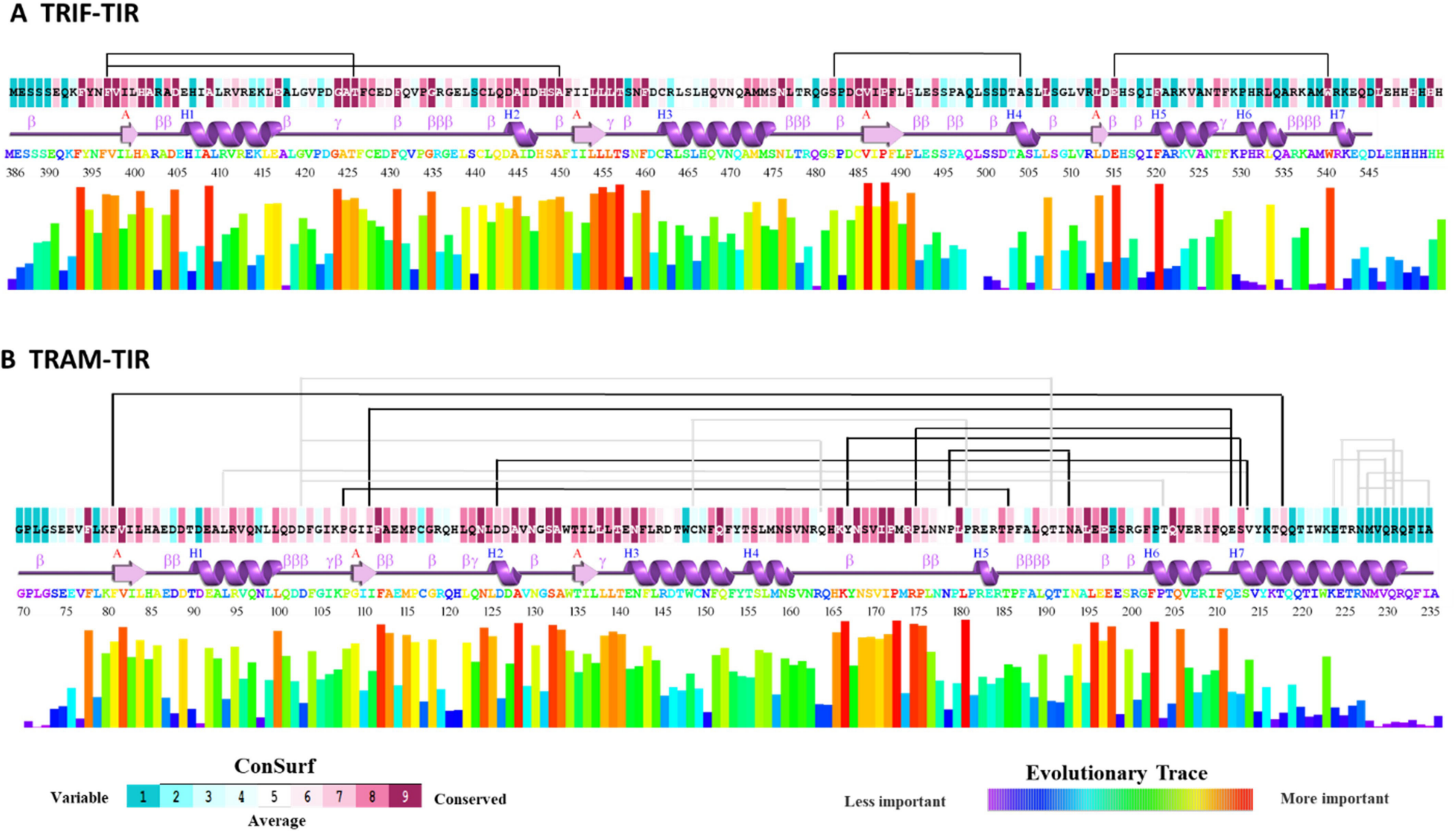
First color plot shows the ConSurf result for degree of conservation on a scale of 1 to 9 among the orthologs. Below is the secondary structure of the TIR domain of the corresponding protein. Then, the colored representation of amino acid sequence and the histogram shows the result from the evolutionary trace method. Above all these, the connecting lines between the amino acid shows the coevolving pairs as predicted from visualCMAT. **(A)** TRIF–TIR data being depicted on PDB: 2M1X, **(B)** TRAM–TIR data depicted on PDB: 2M1W.

Both adaptor proteins TRAM and TRIF contain the TIR domain that facilitates the homotypic interactions leading to signal transduction (8). To identify the TRAM–TIR and TRIF–TIR complex formation among orthologs, we selected few representative sequences to compare detailed structural interaction with reference to *H. sapiens*. We chose representative organisms across various taxonomical orders ranging from the common ancestor of both adaptors in Chondrichthyes, to representatives from Amphibians, Cryptodira, Crocodylia, Bifurcata, Aves, and Mammalia. These orthologs were carefully chosen to have well-defined domain annotation of adaptors. Prior to focusing on these cases, we compared and analyzed all the available sequences of TRAM and TRIF adaptors. As the NMR structure of the TIR domain was available for both TRIF and TRAM proteins in PDB (2M1X and 2M1W), we were interested to investigate the structural significance of conserved residues among the orthologs (41). The ET method was used to rank functional and structurally important residues among orthologs (19). A higher score represents sequence position variation among distant orthologs and a lower score represents variation among close orthologs. This prediction was also extended to ConSurf analysis to substantiate the degree of conservation of residues and map it onto the protein structure (18). Coevolving residues were also predicted using visualCMAT (20). These results are shown by mapping onto the respective secondary structure from PDBsum in **Figure 2** (42).

From these results, we were able to find that the ET analysis goes in parallel with the ConSurf conserved residue analysis. The result from visualCMAT shows many pairs that have sequences from both highly conserved and variable regions. We found four pairs predicted for TRIF–TIR, and all the residues involved were highly conserved among orthologs. These pairs include F397–T426, F397–A450, S482–A504, and E515–W540. Interestingly, F397 was found to coevolve with T246 as well as A450 (**Figure 2A**).

In addition to TRIF–TIR coevolving residue pairs, 18 pairs were predicted to coevolve among TRAM orthologs. From these 18 residue pairs, we highlighted the highly conserved residues of coevolving pairs (**Figure 2B**). These were F81–Q218, P108–P186, I111–E212, D126–V214, Y167–S213, P175–E212, and P179–N193. Among these, E212 was seen to coevolve with I111, as well as P175. Besides that, P108, I111, and D126 were near the BB loop regions, so they might have some significant changes in protein function. The residue Y167, which is important for response to LPS by phosphorylation, was also found to be coevolving with S213 (9). Among these pairs, E212, S213, and V214 all seem to coevolve, indicating that this region may also be of some functional relevance. Apart from these predicted important residues, mutagenesis studies show that P116H, C117H, Y154F, and Y167F are also significant for protein functioning (9–11). It will be interesting to investigate the role of these evolving residues and check for their functional role or if it interrupts protein stability.

Furthermore, the complete list of the coevolving residues along with other parameters is provided in **Supplementary Table S1**. The other coevolving residue pair combinations from different orthologs at the respective positions along with the frequency of occurrence of such varied amino acids are also shown in **Supplementary Table S1**. For those combinations that had a frequency ≥ 1%, we performed virtual mutations at respective positions on the *H. sapiens* TIR structure and calculated the free energy change (ΔΔ*G* kcal/mol = Δ*G*_mut_ − Δ*G*_wt_) using FoldX 5.0 (21).

In the TRIF–TIR domain, apart from F397–A450, other pairs were mostly coevolving either to neutral or toward the stabilizing end. In the TRAM–TIR domain, most of the mutations have destabilizing or neutral effects, except for D126–T204. While analyzing the sequences of orthologs for coevolving residue pairs, we found that the corresponding phenylalanine at the 81st position of TRAM and the 397th position of TRIF is part of a coevolving pair. This residue (F81 of TRAM and F397 of TRIF) is highly conserved and is found to be destabilized when mutated to another residue as observed in ortholog sequences. Thereby, this residue seems to play an important role in the functioning of TIR in both adaptors. Apart from this, Y167 from TRAM, which is known to be important for phosphorylation, coevolves with S213 and leads to either destabilizing or neutral mutations in orthologous proteins. The details of the free energy change due to a coevolving mutation in TRAM and TRIF is mentioned in **Supplementary Figure S1**.

### 3.2 Important motifs and residue conservation in chosen representative organisms

**Figure 3:**
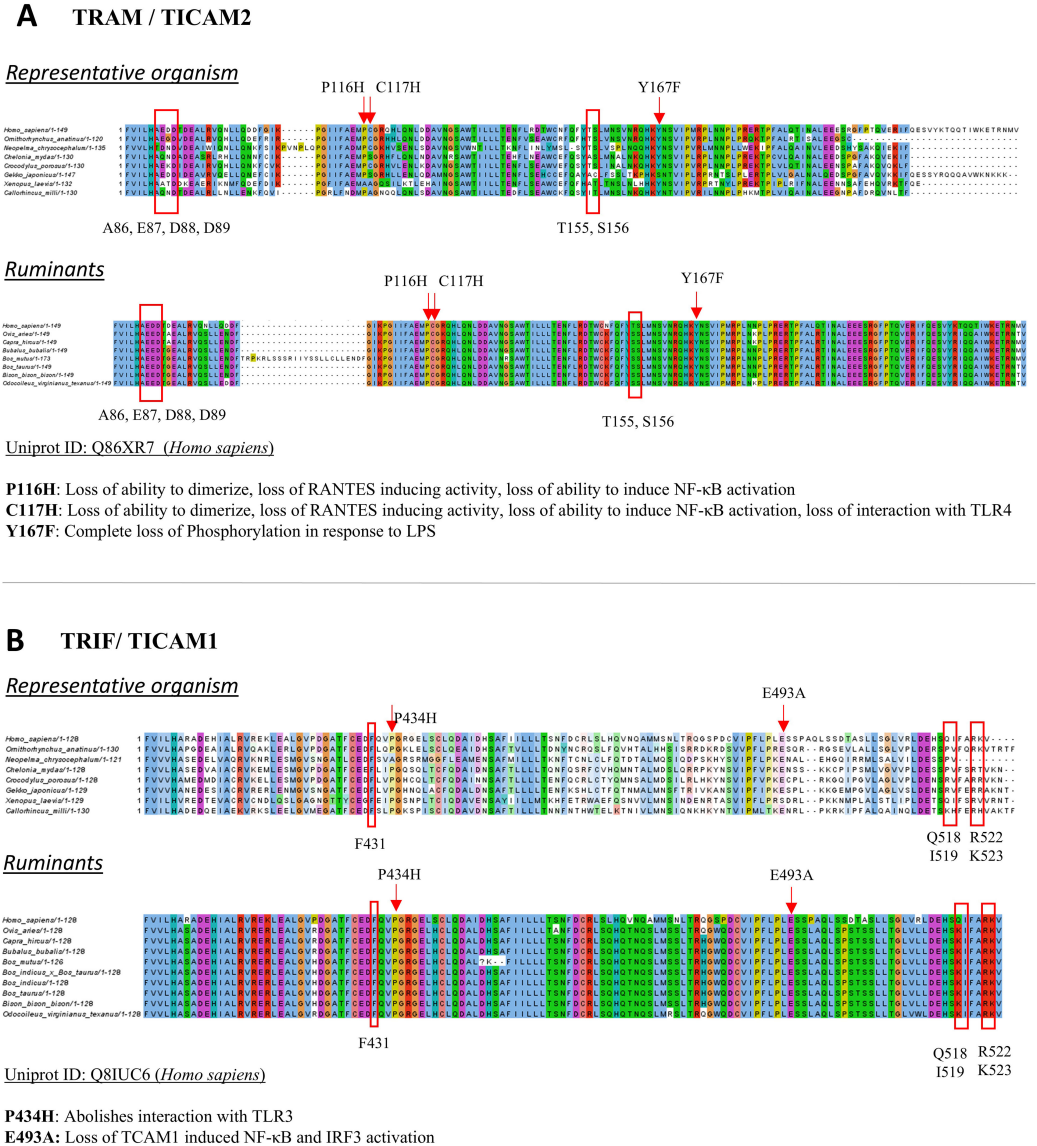
Multiple sequence alignment of **(A)** TRAM and **(B)** TRIF protein of representative organism and ruminants. Key functional residues are highlighted in the alignment.

While focusing on the various taxa representatives, it becomes imperative to observe the key residue patterns, major motifs, and domain conservation to ascertain protein function. We thereby looked at the multiple sequence alignment of TRAM and TRIF proteins in representative organisms and mapped the key residues, motifs, and other patterns of these proteins, such as the AEDD, PC, TS, and Y sites in TRAM and the QI and RK sites in TRIF (8). We also looked at domain architecture and key myristoylation sites, the TRAF6 binding site motif of the TRAM protein, the pLxIS motif, and TRAF6 binding motifs of the TRIF protein. **Figure 3** shows the conservation pattern of the key residues in the representative orthologs and ruminants. We also observed the conservation pattern in the MyD88 and TIRAP protein to validate the existence of the MyD88 signaling pathway in orthologs (**Supplementary Figure S2**).

As both the MyD88-dependent and -independent pathways are part of TLR4 signaling in humans, we searched for lines of evidence of these pathways in representative organisms using the KEGG database (43).

We found that D89 from the AEDD site is conserved in all representatives in the case of TRAM orthologs. However, the key residues P116 and C117 were both mutated to alanine in the case of *X. laevis*, which may abrogate the TRAM-mediated signaling in these organisms. Additionally, Y167 is mutated to serine, in *G. japonicus*, although this may still function as a phosphorylation site. In case of ruminants, we see an insertion for the *B. mutus* sequence, and this can be attributed to it being a predicted sequence. A better-quality genome might help to solve this ambiguity. In addition, we observe that the T155 sequence in ruminants are not well conserved but mutated to serine at the same position; this still might maintain the function as both amino acids are polar in nature.

In the case of TRIF orthologs, the F431 residue was found to be well conserved in each representative and ruminants, thereby ensuring the interaction of TRIF–TIR with TRIF–NTD in an autoinhibited stage (15). The E493 residue is mutated to serine in case of *X. laevis*. Q518 and I519 are not well conserved across species but are known to be experimentally important (8). This can be a specific case of *H. sapiens* TRIF interaction with the TRAM residue. A table listing the key residues from the TRAM and TRIF protein is included in **Supplementary Table S6**. Moreover, the conserved motif and the schematic diagram for the TLR4 pathway provides deeper insights into the functions of these adaptors (**Supplementary Figures S3**, **S4**, respectively). Overall, we find a high level of sequence similarity in the case of ruminants (order Artiodactyla; suborder Ruminantia) with the *H. sapiens*. As both belong to the same Mammalia category, we further followed up with *H. sapiens* and an evolutionary farther representative of Mammals (*O. anatinus*).

### 3.3 Modeling human TRAM and TRIF dimers

The intriguing conservation and coevolving residue patterns prompted us to subsequently model the entire TRAM–TRIF complex. Previous studies indicate an interaction between the TRAM dimer and the TRIF monomer (8); other studies suggest that the homodimerization of TRIF is important for interferon-β production (29, 30).

We employed several approaches to model the TRAM and TRIF dimeric, trimeric, and tetrameric models (details in Materials and Methods). Both dimers were modeled using sequences, docking, HM, and guided docking. The selection of the best TRAM dimer was based on a previous study (22) and the most stable pose, where the dimer remained stable after MD was selected as the best dimer pose.

**Figure 4:**
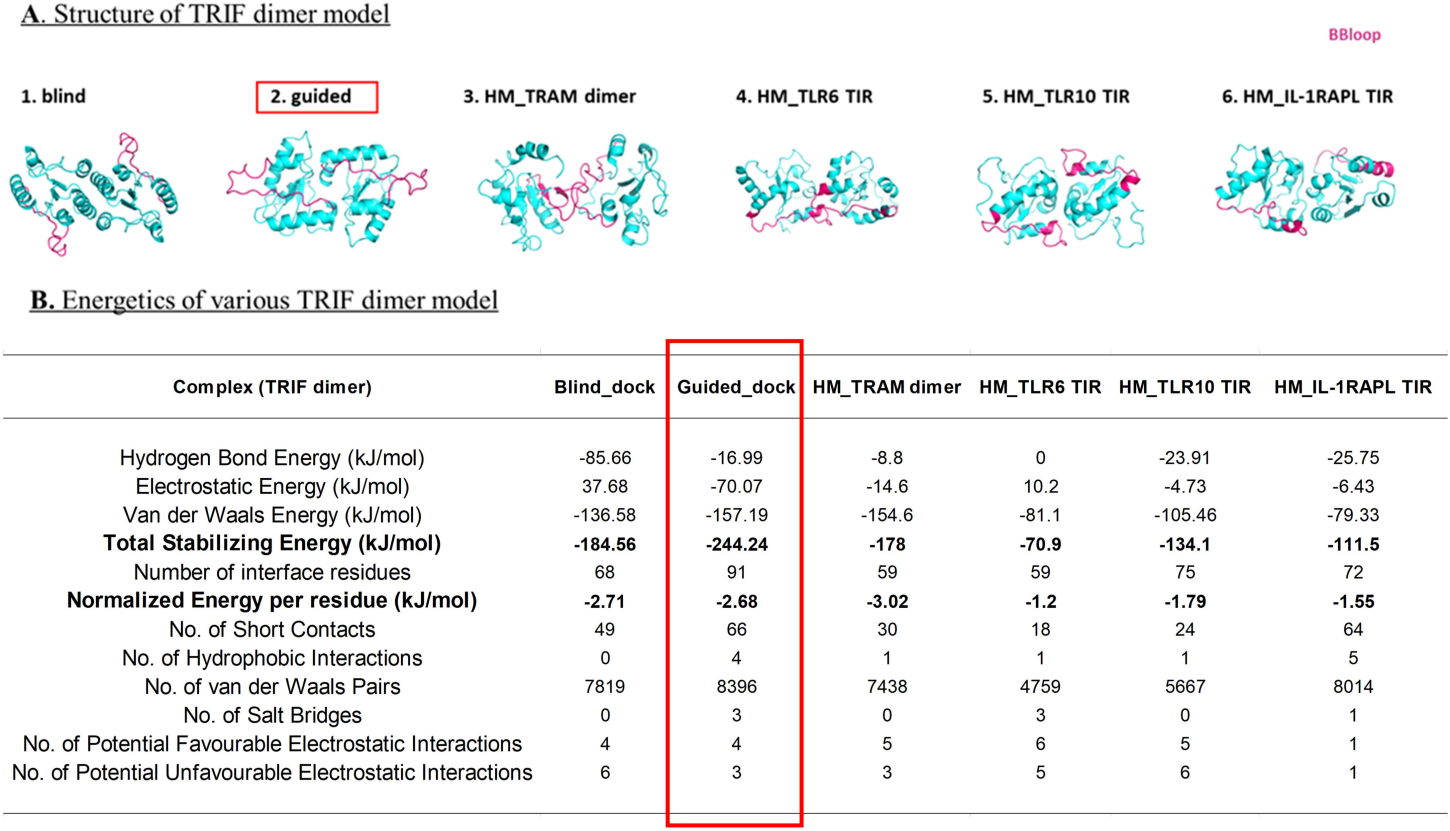
**(A)** Dimer model of TRIF using different methods; BB loop represented in pink color. **(B)** Energetics of each dimer model calculated using PPCheck (27).

In the case of the TRIF dimer, we used blind and guided docking (by BB loop) and HM modeling based on templates of the TRAM dimer (as obtained from stabilized MD), TLR6-TIR, TLR10-TIR, and IL1-RAPL TIR structure. We analyzed the position of the BB loop and calculated the energetics of these models using PPCheck (27). The models with their energy values are shown in **Figure 4**. The dimer model with guided docking displays highly stabilizing energy and comparable normalized energy per residue as compared to other models. Moreover, the position of BB loop at the interface and the HM TRAM dimer also seemed more favorable in the guided docking.

### 3.4 Modeling of human TRAM–TRIF trimer and tetramer complexes

**Figure 5:**
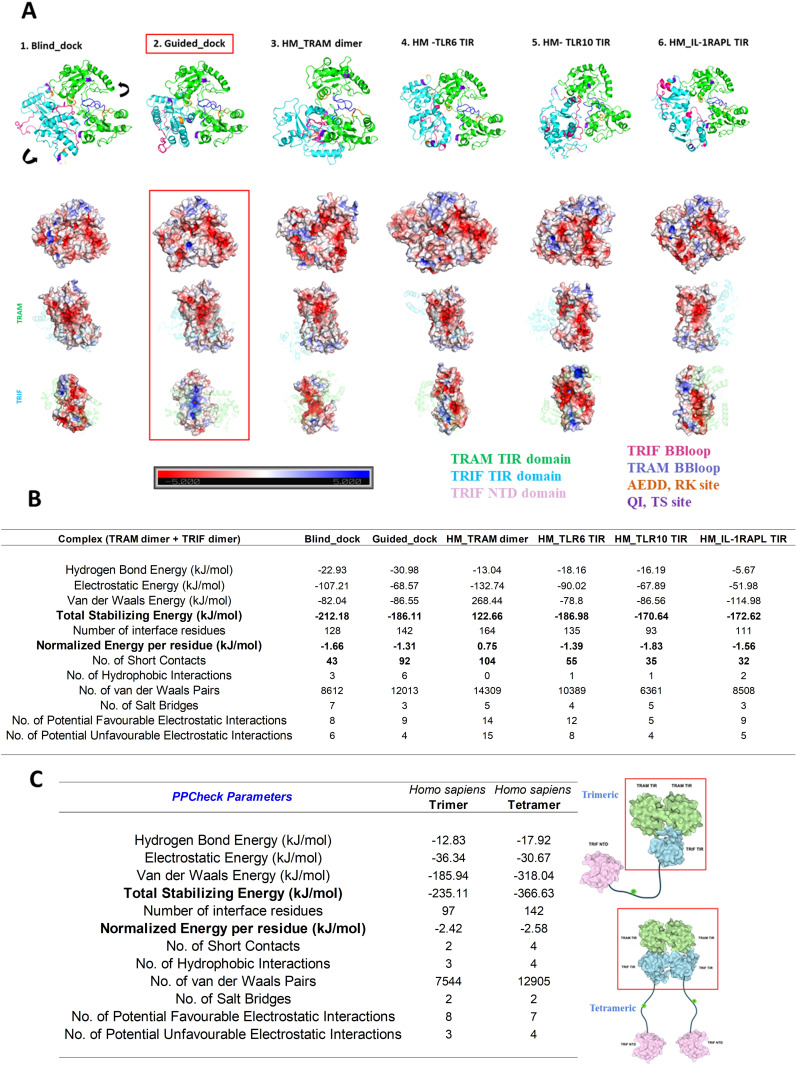
**(A)** Multiple approach for the tetramer model of *Homo sapiens* TRAM and TRIF dimer. The key residues important for interaction are highlighted in different colors. The electrostatic potentials of the dimer interface are also shown in the surface diagram. The color notation of protein in **Figure 4A** is as mentioned: TRAM–TIR dimer in green, TRIF–TIR dimer in sky blue, TRIF BB loop in pink, TRAM BB loop in blue, AEDD, RK site in orange and QI, TS site in purple. **(B)** Energetics of various tetrameric models. **(C)** Energetics of the 200th ns frame molecular dynamic structure trimeric and tetrameric model of the TRAM–TRIF complex of *Homo sapiens* for the best performed model.

We used various modeling techniques such as HM, HADDOCK, HDOCK, and AlphaFold to construct the trimeric and tetrameric protein complexes. We compared the models obtained from these techniques based on the energy values calculated using PPCheck, BB loop positions, and key residue positions. We found that the model generated using HDOCK docking stands out well in terms of satisfying all the validation parameters. Details of the mode of TRAM–TRIF trimeric and tetrameric complexes are provided in Materials and Methods. We subjected both the modeled complexes to 200 ns of MD simulation and compared the energies of the final frame structure of both the complexes. We found that the tetrameric complex is more stable than the trimeric complex. The details of the energies of each trimeric and tetrameric models of representative organisms are included in **Supplementary Material 2** (**Supplementary Tables S2**, **S3**, respectively). Next, we examined the electrostatic interaction between TRAM–TIR and TRIF–TIR in the modeled complex and observed that the tetrameric model has complementary potentials at the interface, which further explains the higher stability of the complex. **Figures 5A, B** show the various possible tetrameric models and final frame energies of the tetrameric model. **Figure 5C** also highlights the energy values of the final frame structure of both the trimeric and tetrameric model.

We observed that the second model, based on the guided docking, performed best in terms of energy and showed complimentary electrostatic patterns (**Figures 5A, B**). **Figure 5C** shows the comparative energy between trimeric and tetrameric models, and we found that the total stabilizing energy for the tetrameric model is higher than the trimeric model, suggesting that it has higher stability.

### 3.5 Normal mode analysis to decipher the residue interaction network

**Figure 6:**
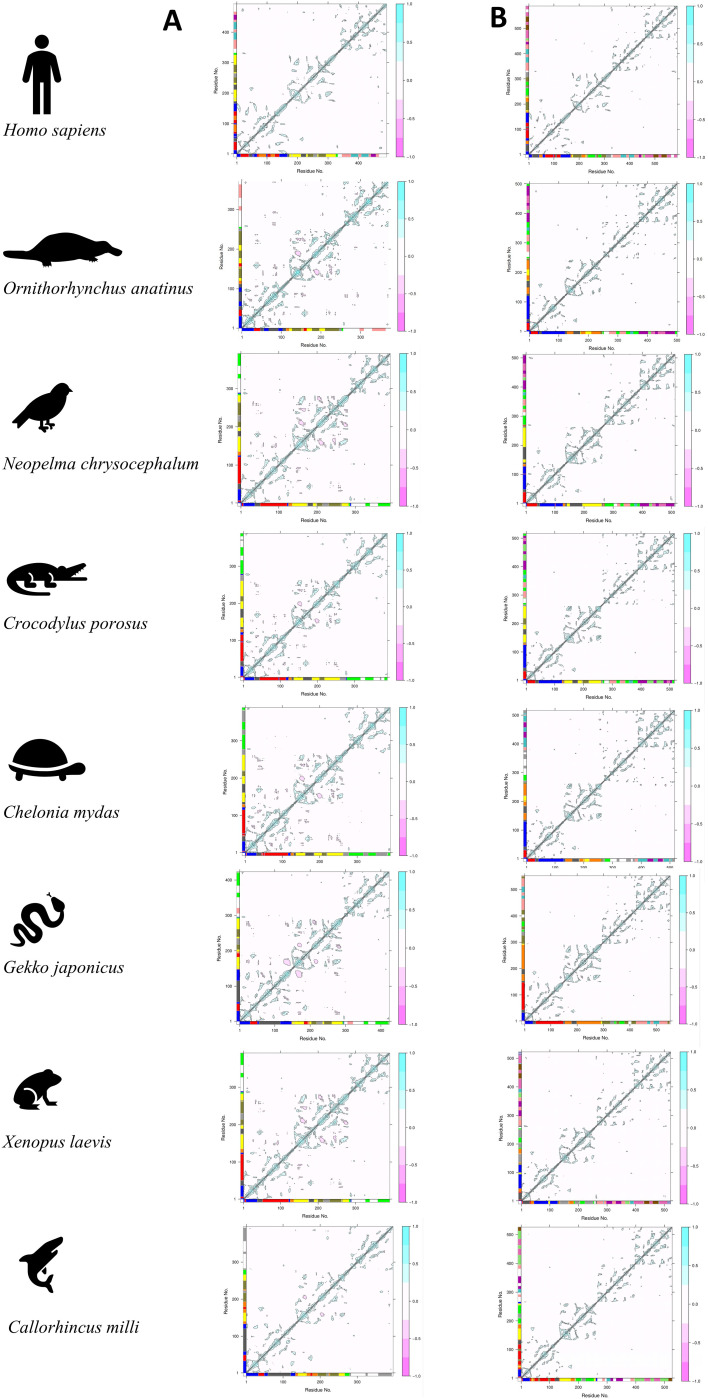
The figure above shows the residue interaction plot of **(A)** the trimer complex (TRAM dimer interacting with TRIF monomer) and **(B)** the tetramer complex (TRAM dimer interacting with the TRIF dimer complex).

Next, we performed NMA on the modeled trimer and tetramer complex of TRAM and TRIF for each representative organism. We made the complex from the orthologs using HDOCK using key residues based on guided docking (25). **Figure 6** shows the residue interaction plot for representative organisms in the trimeric and tetrameric complexes. The residue network plots for trimeric and tetrameric complexes from representative organism are shown in **Supplementary Figure S5**.

We observed a lot of negative interaction in the second chain of TRAM proteins of the trimeric complexes (**Figure 6A**) in all organisms other than *H. sapiens* (shown in pink). This explains the instability of the complex. In the case of the tetrameric complex (**Figure 6B**), only the positive interactions were seen across both the chains of all the organisms (shown in cyan). Meanwhile, when we compared the residue clustering pattern in case of the trimer (**Supplementary Figure S5A**), we did observe a similar trend across *N. chrysocehalum*, *C. porosus*, *C. mydas*, *X. laevis*, and, to some extent, *G. japonica*. This follows a similar trend as with the known evidence of various adaptor proteins involved in TLR4 signaling. From the residue clustering pattern of the tetramer (**Supplementary Figure S5B**), we did not observe any cross interaction between TRAM and TRIF proteins in the case of *G. japonica* and *C. milli* (Australian ghostshark) from the NMA, whereas in other cases, there exist connecting clusters between TRAM and TRIF proteins.

### 3.6 MD analysis to map the dynamic residue network

**Figure 7:**
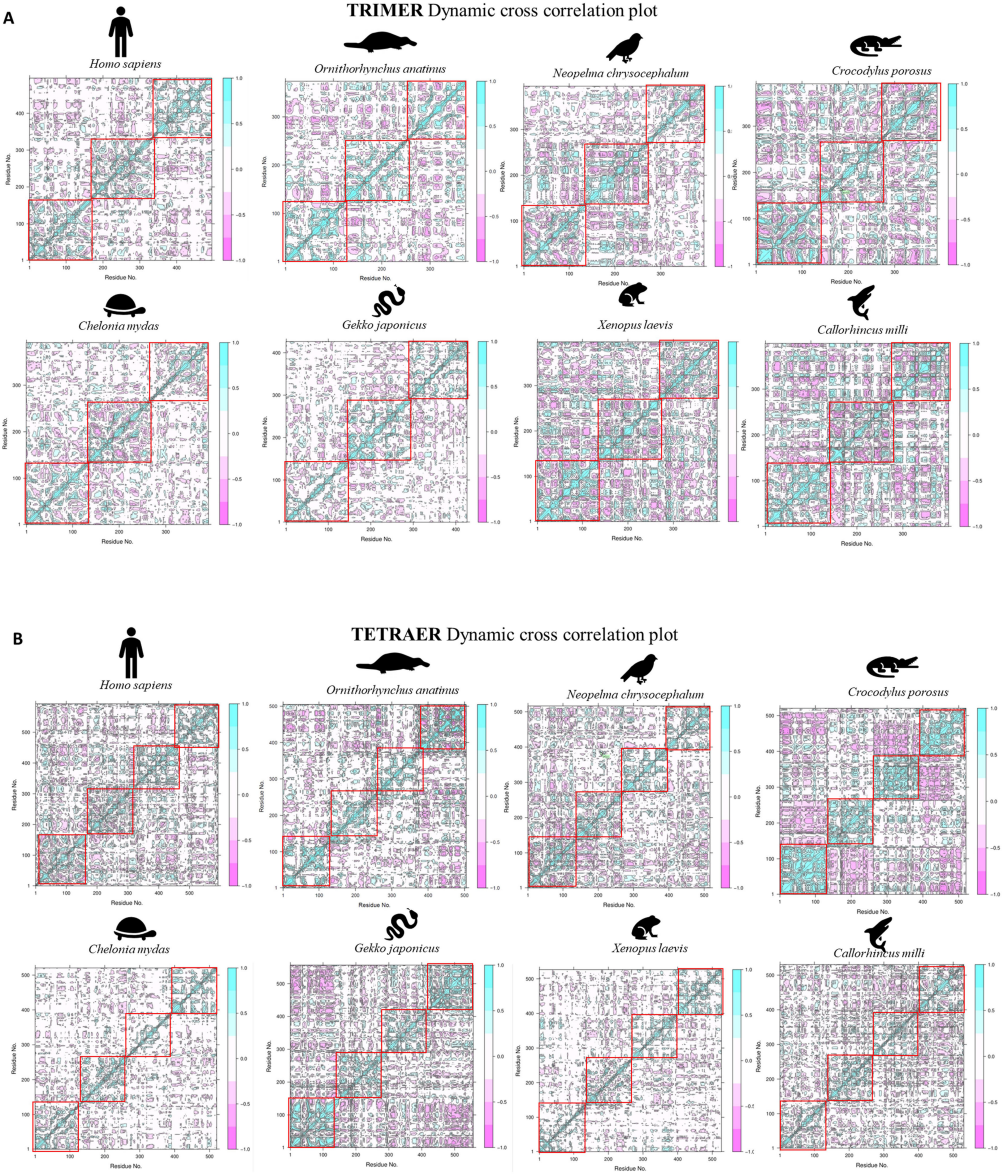
Dynamic cross-correlation plot for TRAM–TRIF **(A)** trimer and **(B)** tetramer complexes of representative organisms. The highlighted red boxes represent the intrachain interactions across the MD trajectory. The strength of the positive interaction is shown in cyan color and negative interaction is shown in pink color.

We ascertained the dynamic trend for the modeled protein complexes using MD trajectory. A 200-ns MD run was compared for the various trimeric and tetrameric complexes of the representative organisms. We used the trajectory further to plot dynamic cross-correlation matrix across the complete length of the protein (**Figure 7**).

On observing the plots, we deciphered that the tetrameric complex shows denser plots, indicating stronger interactions. The intensity of positive interaction (shown in cyan) is found to be higher in the case of the tetrameric complex. This points towards a higher possibility of tetrameric complexes of the TRAM and TRIF dimer. Interestingly, we observed lesser interaction in the case of *C. mydas* and *X. laevis*, which hints at less stable complexes in these cases. Additionally, we compared the root mean square deviation (RMSD), root mean square fluctuation (RMSF), and conservation pattern of the secondary structure of the protein across the trajectory (**Supplementary Figures S6**, **S7**, respectively).

Furthermore, using the centrality analysis, and information from the literature, we measured various centrality measures of these complexes (**Supplementary Tables S4**, **S5**). We compared the BC, being one of the measures of important nodes, between models from representative organisms for the key hotspot residues (TRAM: A86, E87, E88, D89, T155, and S156; TRIF: Q518, I519, R522, and K523), important BB loop residues (TRAM: P116 and C117), and phosphorylation site (TRAM: Y167), among different chains of the trimeric and tetrameric complexes of the representative organisms, at the respective homologous residue (8, 9). This analysis also highlights the persistent nature of important residues across organisms for trimeric and tetrameric complexes (**Supplementary Figures S8**, **S9**, respectively).

## 4 Discussion

### 4.1 Tetrameric complex of TRAM–TIR and TRIF–TIR domains

**Figure 8:**
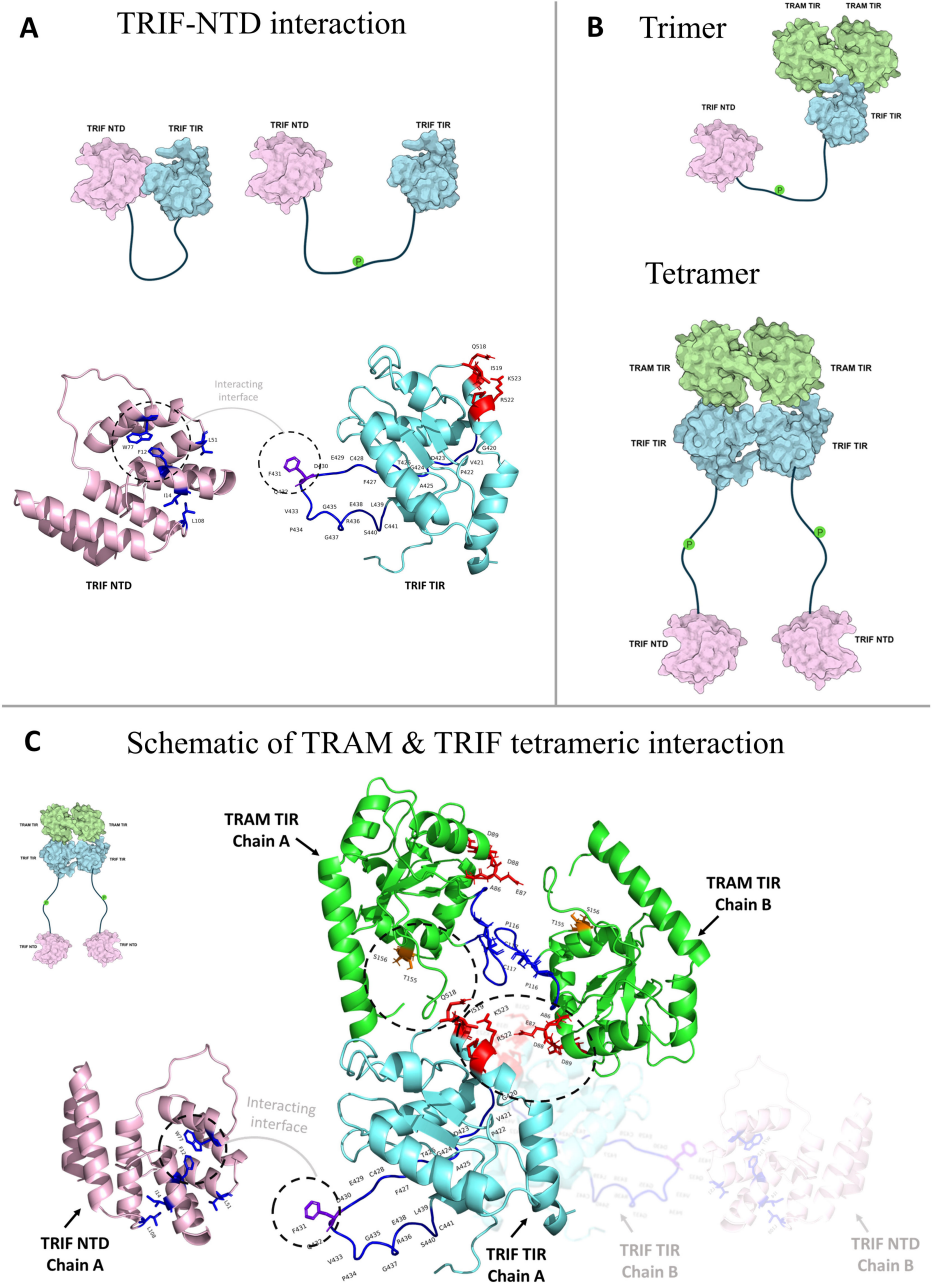
**(A)** Schematic of the interaction between TRIF N-terminal domain (NTD) by the F431 of TIR domain (15). **(B)** Schematic showing the trimeric and tetrameric interaction between TRAM and TRIF. It also shows how TRIF–NTD would change orientation post phosphorylation for interaction with IRF3 further downstream. **(C)** Schematic showing the TRAM dimer interaction through the BB loop residues, and key residues like AEDD, TS with the QI and RK of TRIF protein in a dimeric way. The TRIF–NTD domain is shown separately; it would be connected to the TRIF–TIR by a loop and post phosphorylation separate from TRIF–TIR to interact further with IRF3 (29).

The structural analysis of interactions between the TIR domains of TRAM and TRIF from a few representative genomes shows that the tetrameric form of the TRAM–TRIF heteromer is more stable than the trimeric form, as indicated by the normalized PPCheck energies at the interface. A schematic representing the dimeric–tetrameric complex interaction, keeping in mind the key residues involved in interaction, is shown in **Figure 8**.

### 4.2 Insights from representative organisms

On comparing the various parameters across all the representative organisms and surveying the literature associated with innate immunity, we found some interesting facts about these organisms. We found that *N. chrysocephalum* lacks the CD14 protein that is important for the TRAM-mediated pathway (44, 45). In the case of Avian TLRs, chickens have been studied widely but they lack the TRAM orthologs like most of the birds (46, 47). However, Aves are known to have developed viral RNA sensing via TRIF-mediated TLR3 by producing IFNβ. Furthermore, *N. chrysocephalum* lacks the TRAM myristoylation motif and TRAF6 binding motif, but has conserved serine in the pLxIS motif. It also has a RHIM motif that is important for TRIF-induced apoptosis and also contributes towards TRIF-induced NF-κB production (13, 48). *C. porosus* also lacks the CD14 protein and, like Aves, uses TRIF-mediated TLR3 signaling to sense viral RNA, by producing IRF3 and IRF7 (49). However, unlike Aves, *C. porosus* has both myristoylation motif and TRAF6 binding motif in the TRAM protein and conserved serine in the pLxIS motif of TRIF. TRAM in *C. mydas* also lacks the CD14 and uses TLR3-mediated signaling to sense viral RNA (50). Like *C. porosus*, it has conserved motifs and key residues. In the next taxon representative, *G. japonicus*, the CD14 protein is missing. However, the presence of an alternate pathway recognizing LPS was found to be evolving at this level and positive selection was seen in the TLR3 and TLR4 protein of reptiles (51, 52). Moreover, it has conserved TRAM and TRIF motifs, but these do not interact, as observed through the MD study. The amphibian representative *X. laevis* similarly lacks the CD14 protein and the myristoylation motif on TRAM that is crucial for its localization to the plasma membrane (12). In addition to these, while studying the oldest common ancestor, *C. milli*, the TRAF6 binding motif of TRAM protein was missing, but the myristoylation motif of TRAM and TRIF’s motif remain conserved. Although the interaction study shows distinct clusters for TRAM and TRIF proteins in this taxa, the absence of key residues suggests that the TRAM–TRIF-mediated signaling might be evolving (53). A schematic representing the conserved motif is included in **Supplementary Figure S3**.

Even before the Chondrichthyes (*C. milli*), the ancestor of TRAM and TRIF proteins was observed in Leptocardii (*Branchiostoma belcheri*), also known as amphioxus (7). The evidence of the emergence of the MyD88-independent pathway in amphioxus by discovery of a novel TIR adaptor referred to as bbtTICAM has widened the scope of the TRAM–TRIF-mediated pathway in invertebrates. Even though the bbtTICAM activates NF-kB in a MyD88-independent manner via the interaction with TRIF’s RHIM domain, it fails to induce the production of the TRAM–TRIF-mediated type I IFNs (54). As per the computational analysis of the common ancestor of both the adaptor proteins TRAM and TRIF, we observe that *C. milli* lacks the key residues of TRAM that may similarly hinder the type I IFN-mediated signaling. The conservation of key functional residues in TRIF points toward the presence of MyD88-independent NF-kB activation by the TRIF’s RHIM domain, which involves TLR3.

### 4.3 Highlights of NMA, MD, and residue network analysis

To observe the biomolecular motions from the NMA, we find more negative interaction in trimers, suggesting antagonistic relationships as they highlight the anti-correlated motion. These interaction patterns were also seen to be persistent in *N. chrysocephalum*, *C. porosus, C. mydas*, and *X. laevis*. On analyzing the residue clustering pattern from these correlation plots, we observe a complete dissociation of TRAM and TRIF cluster residues, suggesting a loss of interaction. Although these analyses were done on the static model, these models were further subjected to MD simulation to understand the dynamics of the trajectory.

While comparing both the trimer and tetramer complexes from representative organisms after an MD simulation of 200 ns, we find that overall tetrameric complexes are more stable and compact with lesser RMSD (**Supplementary Figure S11**) than the trimeric ones. This further supports our energetics calculation showing higher stability for the tetrameric model.

The MD trajectory analysis from the dynamic motion of residues shows that the intrachain region shows weaker interaction in most cases. This would be possible as TRAM and TRIF undergo interaction only during signaling, and might be transient in some cases. However, we also find weaker interactions in the case of *C. mydas* and *X. laevis*. A strong interchain negative interaction was observed in the case of *G. japonicus* and *C. porosus*. These negative interactions represent the anti-correlated motions. These might have some structural and functional significance, assisting in allosteric regulation that may assist in achieving a specific conformation state or biological function.

## 5 Conclusions

The sequence and structural patterns of TIR domains of TLR4 adaptors (TRAM and TRIF proteins) among representative orthologs across the tree of life (7) provide a bird’s eye view of the evolutionary trajectory of TLRs. Our modeling approach is an attempt to decipher the possible interaction mechanism between TRAM and TRIF’s TIR domain. While comparing the dynamics of these complexes, we also observed that the stability of the tetrameric complexes is higher than the trimeric complexes. This study has also focused on key residues to examine the persistent nature of interactions across the simulations. However, to comprehend the development of innate immunity in non-mammal species as well as the fully functional TRAM-mediated signaling pathways across diverse taxa, it is crucial to investigate and emphasize such pathways.

In this paper, we have employed modeling and MD of TIR assemblies in the TLR4 pathway to show that the conservation of functional motifs or the crosstalk between them might be affected in the primitive TIR adaptor domains. Hence, either the MyD88-dependent pathway or the TLR3–TRIF-mediated pathways might be operating in the ancient TRAM adaptors, such as those in *B. belcheri* (Amphioxus) and *C. milli* (Ghost shark).

Besides TLRs, the presence of other PRRs in humans is also accountable for innate immunity. These include the nucleotide-binding domain leucine-rich repeat/NOD-like receptors (NLRs), RIG-I-like receptors (RLRs), and C-type lectin receptors (CLRs). Their role in the identification of various ligands from different viruses, bacteria, fungi, and other pathogens makes them essential as a first line of defense against these pathogens (55). The evolutionary study of these PRRs across various taxa will shed more light on the defense mechanism of the innate immune pathways of various organisms.

## Data Availability

The original contributions presented in the study are included in the article/**Supplementary Material**. Further inquiries can be directed to the corresponding author.

## References

[1] Stack J Doyle SL Connolly DJ Reinert LS O’Keeffe KM McLoughlin RM. TRAM is required for TLR2 endosomal signaling to type I IFN induction. J Immunol. (2014) 193:6090–102. doi: 10.4049/jimmunol.1401605 PMC4258402 25385819

[2] Kuzmich NN Sivak KV Chubarev VN Porozov YB Savateeva-Lyubimova TN Peri F. TLR4 signaling pathway modulators as potential therapeutics in inflammation and sepsis. Vaccines. (2017) 5:1–25. doi: 10.3390/vaccines5040034 PMC5748601 28976923

[3] Guven-Maiorov E Keskin O Gursoy A VanWaes C Chen Z Tsai CJ. The architecture of the TIR domain signalosome in the toll-like receptor-4 signaling pathway. Sci Rep. (2015) 5:13128. doi: 10.1038/srep13128 PMC4544004 26293885

[4] Akira S Takeda K Kaisho T. Toll-like receptors: critical proteinslinking innate and acquired immunity. Nat Immunol. (2001) 2:675–80. doi: 10.1038/90609 11477402

[5] Roach JM Racioppi L Jones CD Masci AM. Phylogeny of toll-like receptor signaling: adapting the innate response. PloS One. (2013) 8(1):e54156. doi: 10.1371/journal.pone.0054156 23326591 PMC3543326

[6] Fornarino S Laval G Barreiro LB Manry J Vasseur E Quintana-Murci L. Evolution of the TIR domain-containing adaptors in humans: Swinging between constraint and adaptation. Mol Biol Evol. (2011) 28:3087–97. doi: 10.1093/molbev/msr137 21659570

[7] Verma S Sowdhamini R. A genome-wide search of {Toll/Interleukin-1} receptor ({TIR}) domain-containing adapter molecule ({TICAM}) and their evolutionary divergence from other {TIR} domain containing proteins. Biol Direct. (2022) 17:1–14. doi: 10.1186/s13062-022-00335-9 36056415 PMC9440496

[8] Enokizono Y Kumeta H Funami K Horiuchi M Sarmiento J Yamashita K. Structures and interface mapping of the TIR domaincontaining adaptor molecules involved in interferon signaling. Proc Natl Acad Sci U S A. (2013) 110(49):19908–13. doi: 10.1073/pnas.1222811110 PMC3856793 24255114

[9] Huai W Song H Wang L Li B Zhao J Han L. Phosphatase PTPN4 preferentially inhibits TRIF-dependent TLR4 pathway by dephosphorylating TRAM. J Immunol. (2015) 194(9):4458–65. doi: 10.4049/jimmunol.1402183 25825441

[10] Fitzgerald KA Rowe DC Barnes BJ Caffrey DR Visintin A Latz E. LPS-TLR4 signaling to IRF-3/7 and NF-κB involves the toll adapters TRAM and TRIF. J Exp Med. (2003) 198:1043–55. doi: 10.1084/jem.20031023 PMC2194210 14517278

[11] Oshiumi H Sasai M Shida K Fujita T Matsumoto M Seya T. TIR-containing adapter molecule (TICAM)-2, a bridging adapter recruiting to toll-like receptor 4 TICAM-1 that induces interferon-β. J Biol Chem. (2003) 278:49751–62. doi: 10.1074/jbc.M305820200 14519765

[12] Rowe DC McGettrick AF Latz E Monks BG Gay NJ Yamamoto M. The myristoylation of TRIF-related adaptor molecule is essential for Toll-like receptor 4 signal transduction. Proc Natl Acad Sci U S A. (2006) 103:6299–304. doi: 10.1073/pnas.0510041103 PMC1458872 16603631

[13] Kaiser WJ Offermann MK. Apoptosis induced by the toll-like receptor adaptor TRIF is dependent on its receptor interacting protein homotypic interaction motif. J Immunol. (2005) 174(8):4942–52. doi: 10.4049/jimmunol.174.8.4942 15814722

[14] Sato S Sugiyama M Yamamoto M Watanabe Y Kawai T Takeda K. Toll/IL-1 receptor domain-containing adaptor inducing IFN-beta (TRIF) associates with TNF receptor-associated factor 6 and TANK-binding kinase 1, and activates two distinct transcription factors, NF-kappa B and IFN-regulatory factor-3, in the Toll-like r. J Immunol. (2003) 171:4304–10. doi: 10.4049/jimmunol.171.8.4304 14530355

[15] Mahita J Sowdhamini R. Integrative modelling of TIR domain-containing adaptor molecule inducing interferon- β (TRIF) provides insights into its autoinhibited state. Biol Direct. (2017) 12:9. doi: 10.1186/s13062-017-0179-0 28427457 PMC5397763

[16] Pereira M Gazzinelli RT. Regulation of innate immune signaling by IRAK proteins. Front Immunol. (2023) 14:1–14. doi: 10.3389/fimmu.2023.1133354 PMC9972678 36865541

[17] Yamamoto M Takeda K Akira S. TIR domain-containing adaptors define the specificity of TLR signaling. Mol Immunol. (2004) 40:861–8. doi: 10.1016/j.molimm.2003.10.006 14698224

[18] Ashkenazy H Abadi S Martz E Chay O Mayrose I Pupko T. ConSurf 2016: an improved methodology to estimate and visualize evolutionary conservation in macromolecules. Nucleic Acids Res. (2016) 44(W1):W344–50. doi: 10.1093/nar/gkw408 PMC4987940 27166375

[19] Lua RC Wilson SJ Konecki DM Wilkins AD Venner E Morgan DH. UET: A database of evolutionarily-predicted functional determinants of protein sequences that cluster as functional sites in protein structures. Nucleic Acids Res. (2016) 44(D1):D308–12. doi: 10.1093/nar/gkv1279 PMC4702906 26590254

[20] Suplatov D Sharapova Y Timonina D Kopylov K Švedas V. The visualCMAT: A web-server to select and interpret correlated mutations/co-evolving residues in protein families. J Bioinform Comput Biol. (2018) 16(2):1840005. doi: 10.1142/S021972001840005X 29361894

[21] Delgado J Radusky LG Cianferoni D Serrano L. FoldX 5.0: Working with RNA, small molecules and a new graphical interface. Bioinformatics. (2019) 35(20):4168–9. doi: 10.1093/bioinformatics/btz184 PMC6792092 30874800

[22] Verma S Menon R Sowdhamini R. Structural insights into the role of deleterious mutations at the dimeric interface of Toll-like receptor interferon-β related adaptor protein. Proteins Struct Funct Bioinform. (2024) 92(10):1242–58. doi: 10.1002/prot.v92.10 38814166

[23] Van Zundert GCP Rodrigues JPGLM Trellet M Schmitz C Kastritis PL Karaca E. The HADDOCK2.2 web server: user-friendly integrative modeling of biomolecular complexes. J Mol Biol. (2016) 428:720–5. doi: 10.1016/j.jmb.2015.09.014 26410586

[24] Honorato RV Koukos PI Jiménez-García B Tsaregorodtsev A Verlato M Giachetti A. Structural biology in the clouds: the weNMR-EOSC ecosystem. Front Mol Biosci. (2021) 8:1–7. doi: 10.3389/fmolb.2021.729513 PMC8356364 34395534

[25] Yan Y Zhang D Zhou P Li B Huang SY. HDOCK: A web server for protein-protein and protein-DNA/RNA docking based on a hybrid strategy. Nucleic Acids Res. (2017) 45:W365–73. doi: 10.1093/nar/gkx407 PMC5793843 28521030

[26] Jumper J Evans R Pritzel A Green T Figurnov M Ronneberger O. Highly accurate protein structure prediction with {AlphaFold}. Nature. (2021) 596:583–9. doi: 10.1038/s41586-021-03819-2 PMC8371605 34265844

[27] Sukhwal A Sowdhamini R. PPcheck: A webserver for the quantitative analysis of protein-protein interfaces and prediction of residue hotspots. Bioinform Biol Insights. (2015) 9:141–51. doi: 10.4137/BBI.S25928 PMC4578551 26448684

[28] Yan Y Tao H He J Huang SY. The HDOCK server for integrated protein–protein docking. Nat Protoc. (2020) 15:1829–52. doi: 10.1038/s41596-020-0312-x 32269383

[29] Funami K Sasai M Oshiumi H Seya T Matsumoto M. Homo-oligomerization is essential for toll/interleukin-1 receptor domain-containing adaptor molecule-1-mediated NF-κB and interferon regulatory factor-3 activation. J Biol Chem. (2008) 283:18283–91. doi: 10.1074/jbc.M801013200 PMC2440629 18450748

[30] Oshiumi H Matsumoto M Funami K Akazawa T Seya T. TICAM-1, an adaptor molecule that participates in Toll-like receptor 3-mediated interferon-β induction. Nat Immunol. (2003) 4:161–7. doi: 10.1038/ni886 12539043

[31] Baker NA Sept D Joseph S Holst MJ McCammon JA. Electrostatics of nanosystems: Application to microtubules and the ribosome. Proc Natl Acad Sci U S A. (2001) 98:10037–41. doi: 10.1073/pnas.181342398 PMC56910 11517324

[32] Šali A Blundell T. L. Comparative protein modelling by satisfaction of spatial restraints. J Mol Biology. (1993) 234(3):779–815. doi: 10.1006/jmbi.1993.1626 8254673

[33] Grant BJ Rodrigues APC ElSawy KM McCammon JA Caves LSD. Bio3d: An R package for the comparative analysis of protein structures. Bioinformatics. (2006) 22:2695–6. doi: 10.1093/bioinformatics/btl461 16940322

[34] Skjærven L Yao X Scarabelli G Grant BJ. Integrating protein structural dynamics and evolutionary analysis with Bio3D. BMC bioinformatics (2014) 15(1):399. doi: 10.1186/s12859-014-0399-6 25491031 PMC4279791

[35] Hinsen K Petrescu A Dellerue S Bellissent-funel M Kneller GR. Harmonicity in slow protein dynamics. Chemical Physics (2000) 261:25–37. doi: 10.1016/S0301-0104(00)00222-6

[36] Schrödinger. Protein Preparation Wizard | Schrödinger. Schrödinger Release 2018-1 (2018).

[37] Valle JVL Mendonça BHS Barbosa MC Chacham H de Moraes EE. Accuracy of TIP4P/2005 and SPC/fw water models. J Phys Chem B. (2024) 128:1091–7. doi: 10.1021/acs.jpcb.3c07044 38253517

[38] Lu C Wu C Ghoreishi D Chen W Wang L Damm W. OPLS4: improving force field accuracy on challenging regimes of chemical space. J Chem Theory Comput. (2021) 17:4291–300. doi: 10.1021/acs.jctc.1c00302 34096718

[39] Schrödinger Release. Desmond Molecular Dynamics System. Schrödinger LLC (2019).

[40] Sheik Amamuddy O Glenister M Tshabalala T Tastan Bishop Ö. MDM-TASK-web: MD-TASK and MODE-TASK web server for analyzing protein dynamics. Comput Struct Biotechnol J. (2021) 19:5059–71. doi: 10.1016/j.csbj.2021.08.043 PMC8455658 34589183

[41] Protein Data Bank. RCSB PDB: Homepage. Rcsb Pdb (2019).

[42] Laskowski RA Jabłońska J Pravda L Vařeková RS Thornton JM. PDBsum: Structural summaries of PDB entries. Protein Sci. (2018) 27:129–34. doi: 10.1002/pro.v27.1 PMC5734310 28875543

[43] Kanehisa M Goto S. KEGG: kyoto encyclopedia of genes and genomes. Nucleic Acids Res. (2000) 28:27–30. doi: 10.1093/nar/28.1.27 10592173 PMC102409

[44] Jiang Z Georgel P Du X Shamel L Sovath S Mudd S. CD14 is required for MyD88-independent LPS signaling. Nat Immunol. (2005) 6:565–70. doi: 10.1038/ni1207 15895089

[45] Tanimura N Saitoh S Matsumoto F Akashi-Takamura S Miyake K. Roles for LPS-dependent interaction and relocation of TLR4 and TRAM in TRIF-signaling. Biochem Biophys Res Commun. (2008) 368:94–9. doi: 10.1016/j.bbrc.2008.01.061 18222170

[46] Brownlie R Allan B. Avian toll-like receptors. Cell Tissue Res. (2011) 343:121–30. doi: 10.1007/s00441-010-1026-0 20809414

[47] Nie L Cai S Shao J Chen J Chen J. Toll-like receptors, associated biological roles, and signaling networks in non-mammals. Front Immunol. (2018) 9:1523. doi: 10.3389/fimmu.2018.01523 30034391 PMC6043800

[48] Chen S Cheng A Wang M. Innate sensing of viruses by pattern recognition receptors in birds. Vet Res. (2013) 44:82. doi: 10.1186/1297-9716-44-82 24016341 PMC3848724

[49] Sarker S Wang Y Warren-smith B Helbig KJ. Dynamic changes in host gene expression following *In Vitro* Viral Mimic stimulation in crocodile cells. Front Immunol. (2017) 8:1–15. doi: 10.3389/fimmu.2017.01634 29213275 PMC5702629

[50] Nash A Ryan EJ Asia E Asia E. Immunity in sea turtles: review of a host-pathogen arms race millions of years in the running habitat. Animals: Open Access J MDPI. (2023) 13(4):556. doi: 10.3390/ani13040556 PMC9951749 36830343

[51] Priyam M Tripathy M Rai U Ghorai SM. Tracing the evolutionary lineage of pattern recognition receptor homologues in vertebrates: An insight into reptilian immunity via *de novo* sequencing of the wall lizard splenic transcriptome. Vet Immunol Immunopathol. (2016) 172:26–37. doi: 10.1016/j.vetimm.2016.03.002 27032500

[52] Zimmerman LM Vogel LA Bowden RM. Understanding the vertebrate immune system: insights from the reptilian perspective. J Experimental Biol. (2010) 213(5):661–671. doi: 10.1242/jeb.038315 20154181

[53] Wu B Xin B Jin M Wei T Bai Z. Comparative and phylogenetic analyses of three TIR domain-containing adaptors in metazoans: Implications for evolution of TLR signaling pathways. Dev Comp Immunol. (2011) 35(7):764–73. doi: 10.1016/j.dci.2011.02.009 21362440

[54] Yang M Yuan S Huang S Li J Xu L Huang H. Characterization of bbtTICAM from amphioxus suggests the emergence of a MyD88-independent pathway in basal chordates. Cell Res. (2011) 21:1410–1423. doi: 10.1038/cr.2011.156 PMC3193451 21931360

[55] Li D Wu M. Pattern recognition receptors in health and diseases. Signal Transduct Target Ther. (2021) 6:1–24. doi: 10.1038/s41392-021-00687-0 34344870 PMC8333067

