# Supplementary material for: Toll-like receptor 4 pathway evolutionary trajectory and functional emergence
Source: Front Immunol. 2025 Jan 20;15:1494017. doi: 10.3389/fimmu.2024.1494017 (PMC11788365; doi:10.3389/fimmu.2024.1494017)
Supplement: Supplementary file 1 [file DataSheet1.docx]

Supplementary Material 1

#
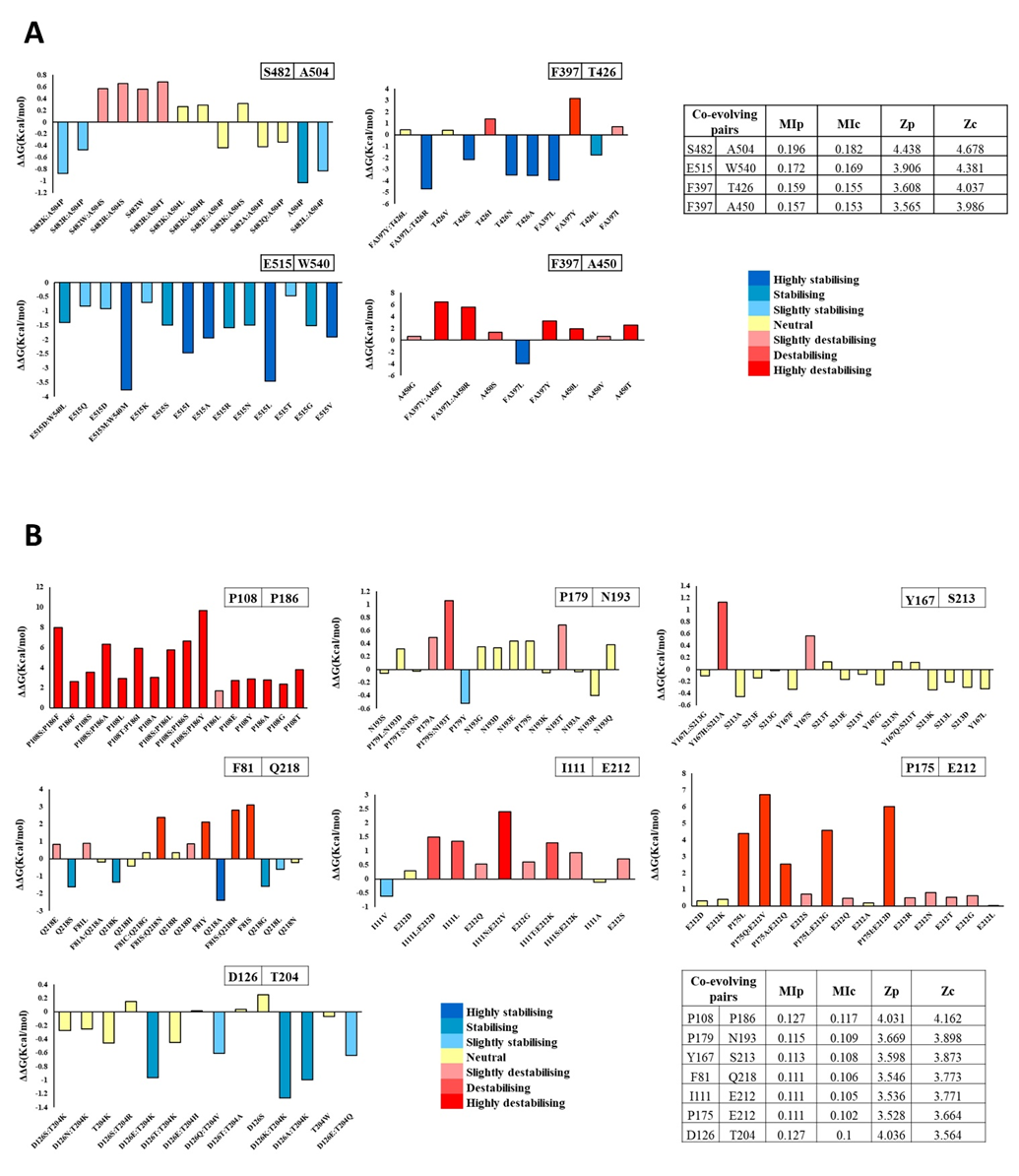
Figure S1

**Figure S1.** A) shows the ΔΔG values from different coevolving pairs of TRIF protein and B) for TRAM protein. The free energy change value is shown by different colours based on stabilising or destabilising nature. Table shows the result from VisualCMAT of coevolving pairs, where MIp and MIc are depicted from mutual information-based statistics and Zp, Zc are the Z score that should be > 3.5 for further consideration. The colour of the bar plot shows the nature of the mutation and is binned into category based on different scores (kcal/mol) as follows: highly stabilising (ΔΔG < −1.84); stabilising (−1.84 ≤ ΔΔG < −0.92); slightly stabilising (−0.92 ≤ ΔΔG < −0.46); neutral (−0.46 < ΔΔG ≤ +0.46); slightly destabilising (+0.46 < ΔΔG ≤ +0.92); destabilising (+0.92 < ΔΔG ≤ +1.84); highly destabilising (ΔΔG > +1.84).

#
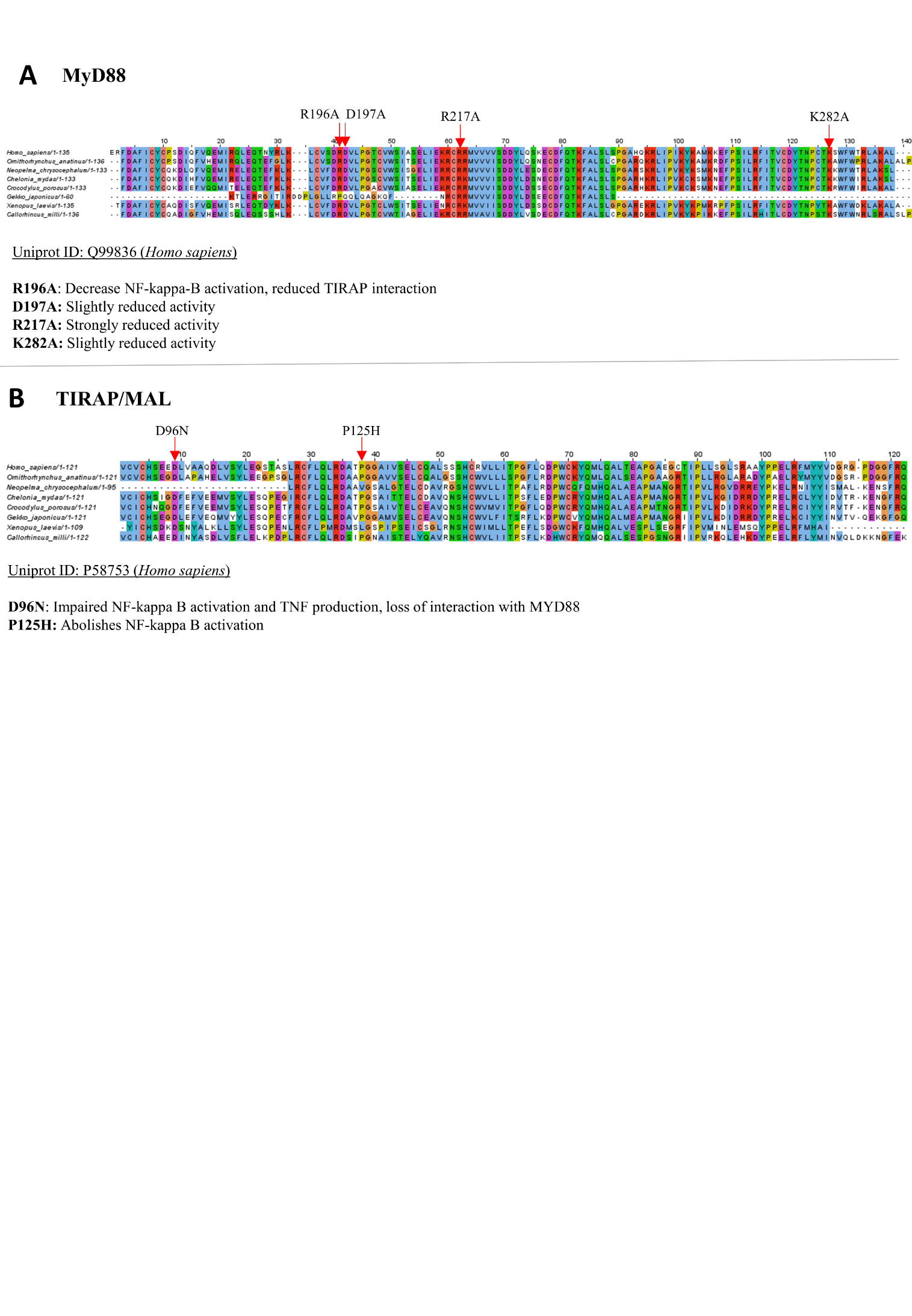
Figure S2

**Figure S2: A)** Sequence alignment of MyD88 protein from representative organisms. The key residues as per *Homo sapiens* Uniprot entry (ID: Q99836) are highlighted with mutational effects. **B)** Sequence alignment of TIRAP protein from representative organisms. The key residues as per *Homo sapiens* Uniprot entry (ID: P58753) are highlighted with mutational effects.

# Figure S3

**
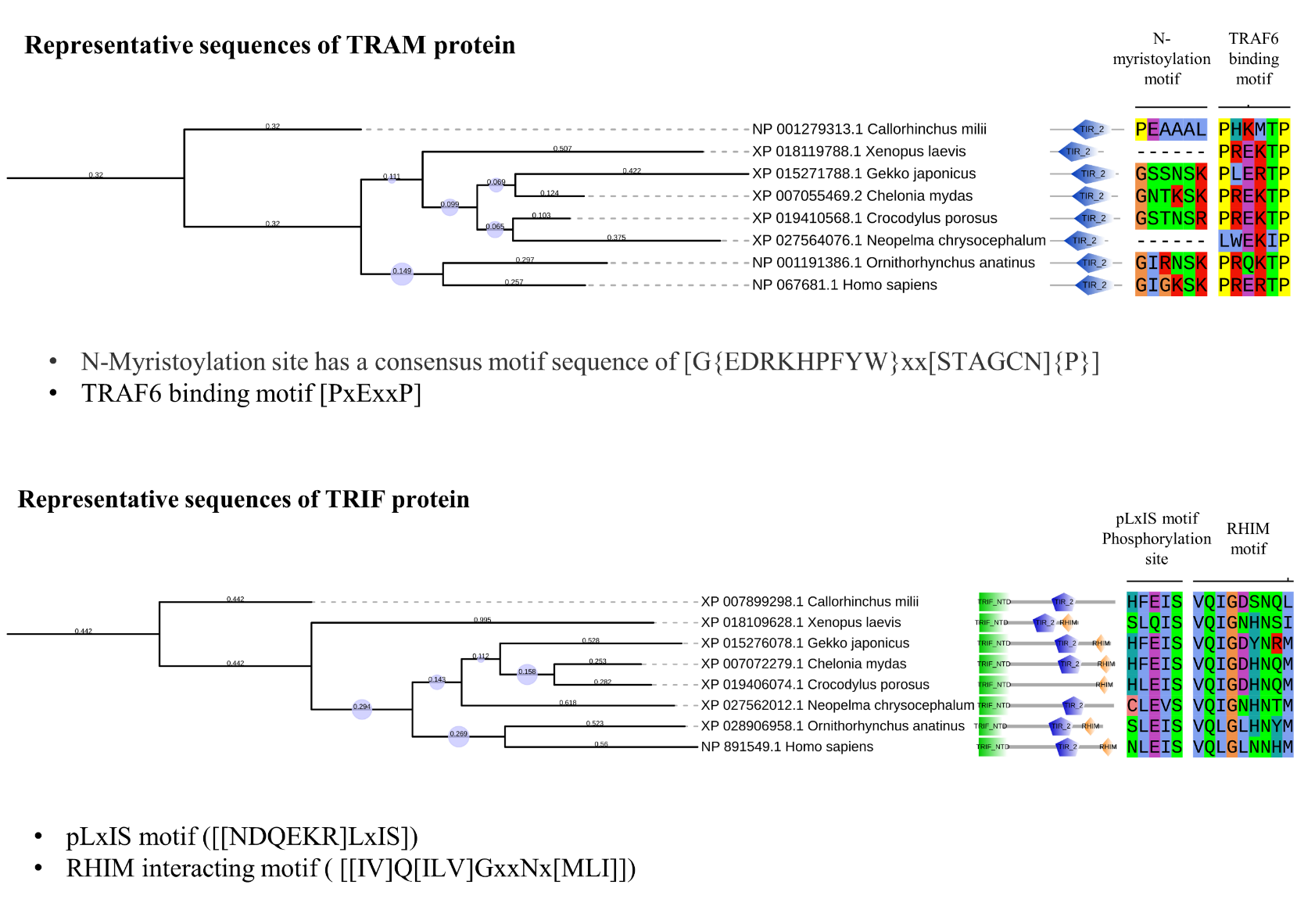
**

**Figure S3:** The phylogeny showing the conserved motifs and domain architectures of representative sequences from **A) TRAM** and **B) TRIF** protein

#
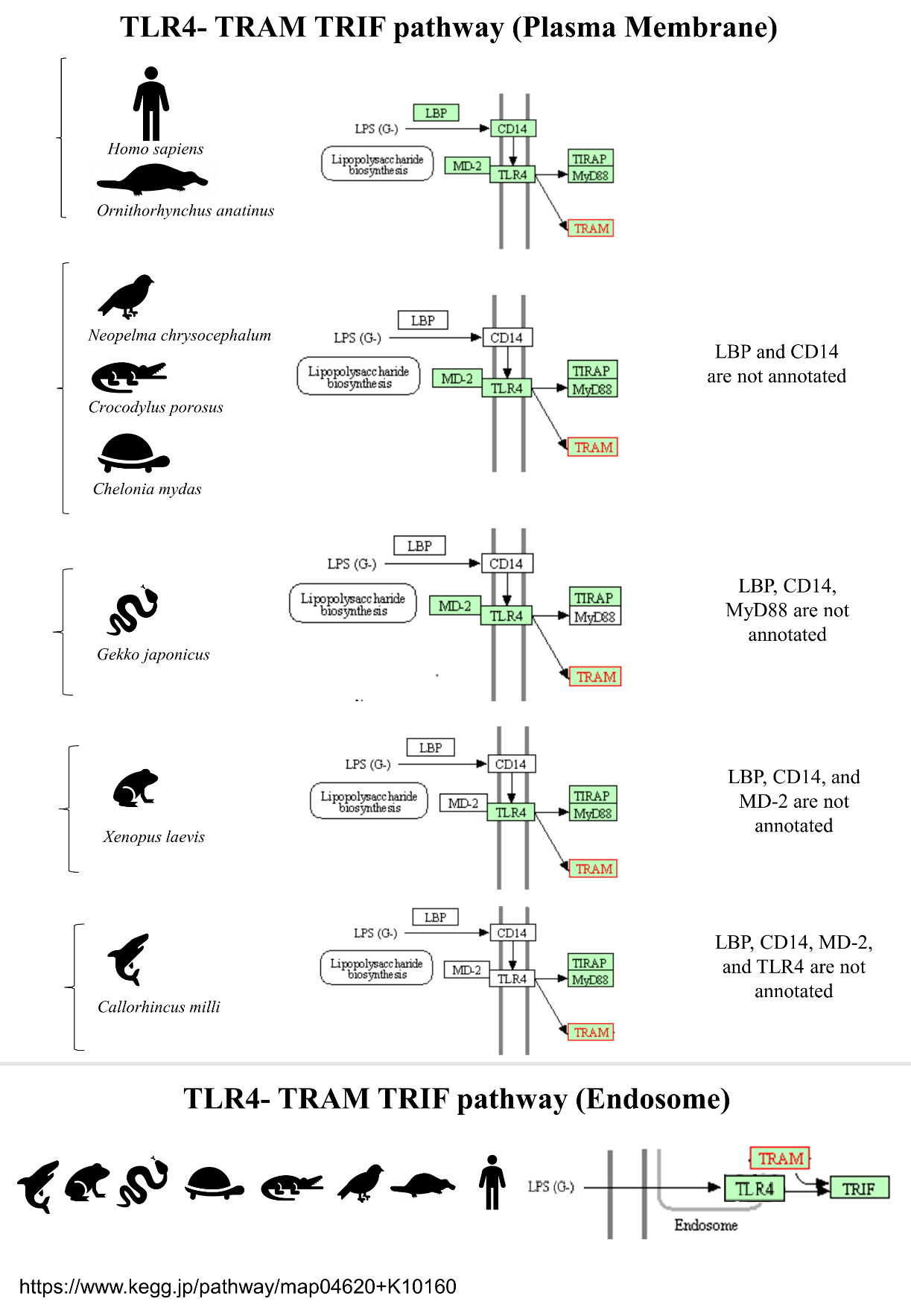
Figure S4

**Figure S4:** The schematic diagram for the TLR4 pathway of representative organisms. The pathways are taken from the Kegg database.

#
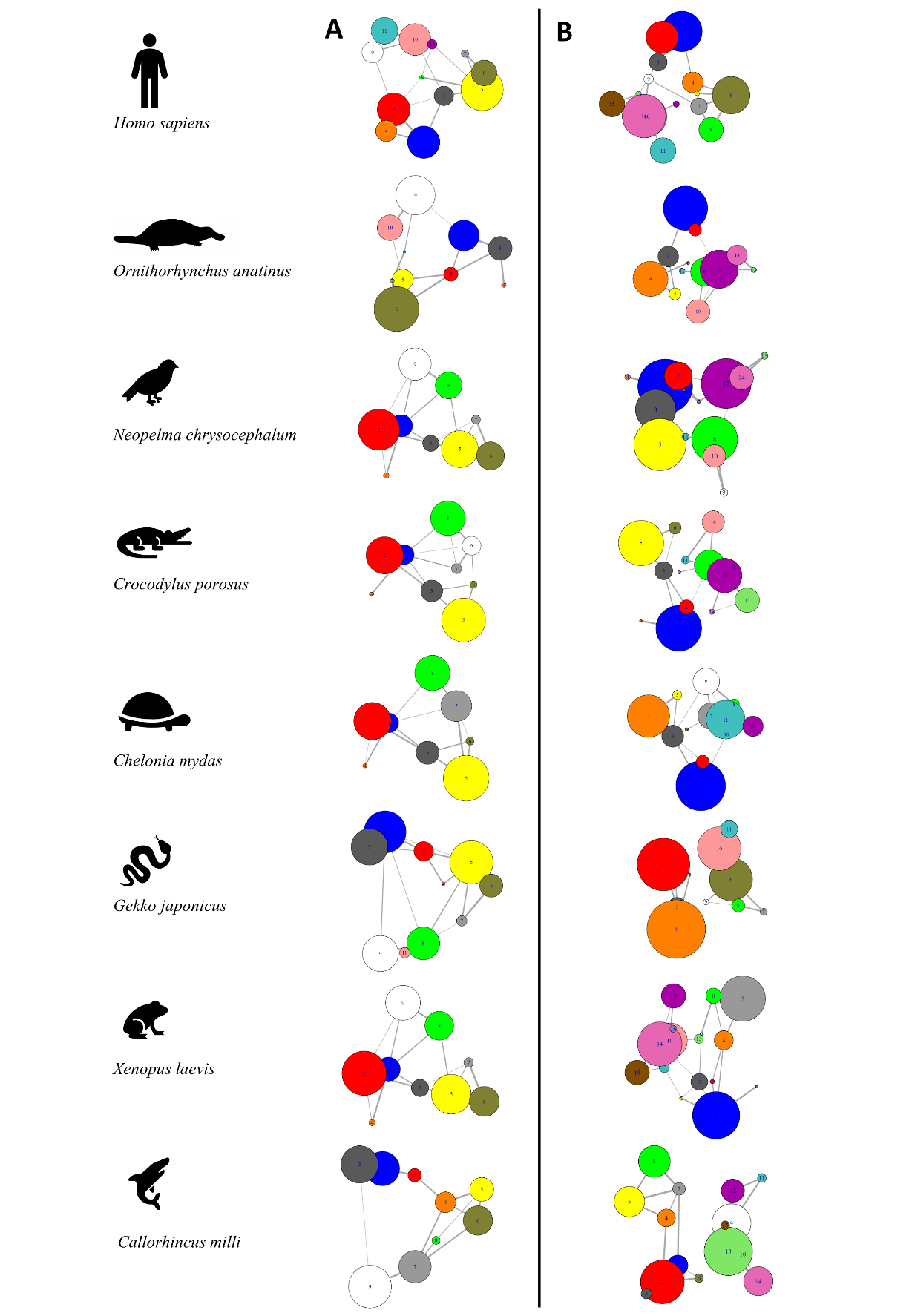
Figure S5

**Figure S5:** The residue network plots for trimer and tetramer complex from the representative organisms.

#
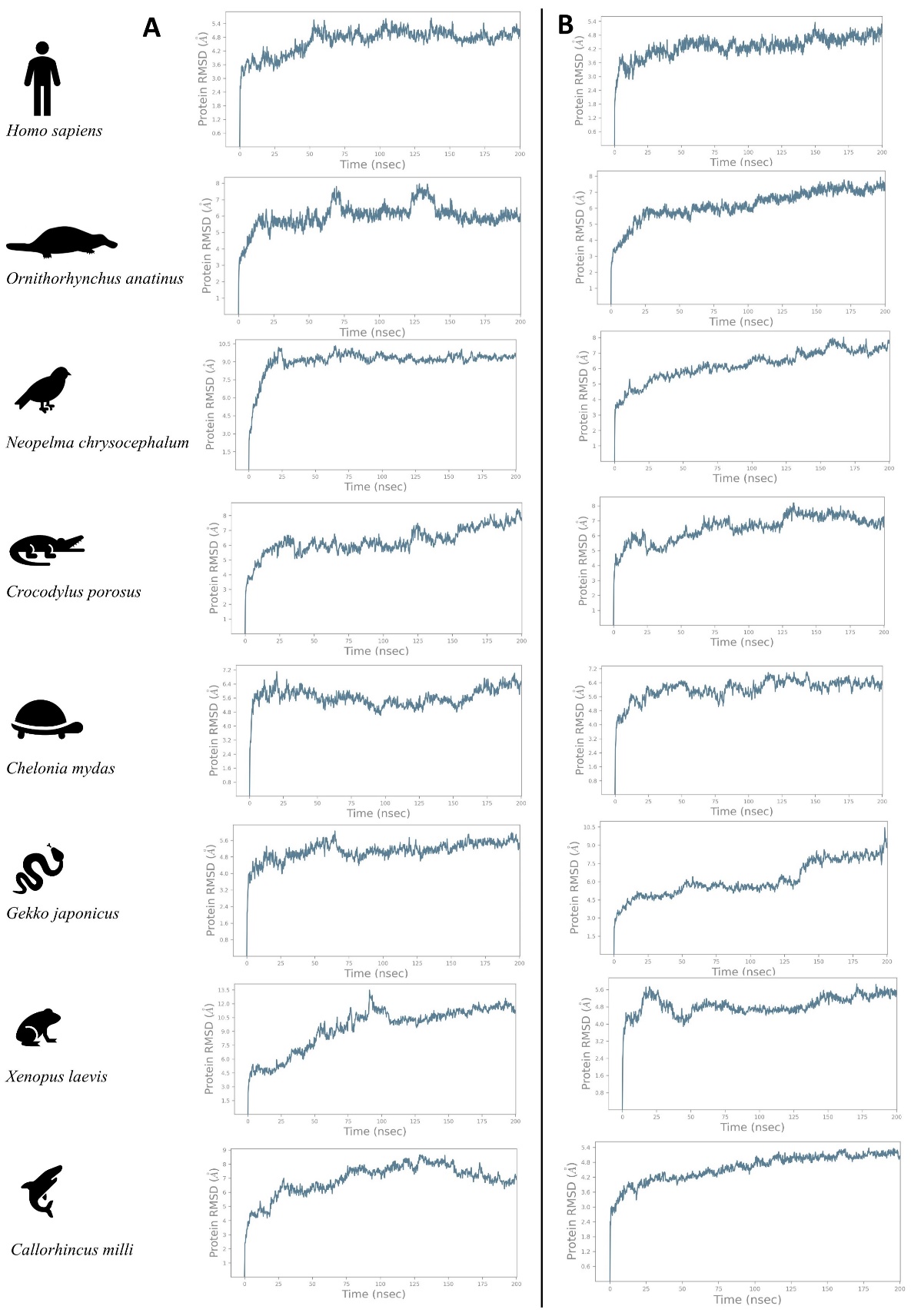
Figure S6

**Figure S6:** The root mean square deviation (RMSD) of the trimeric and tetrameric protein across the trajectory

#
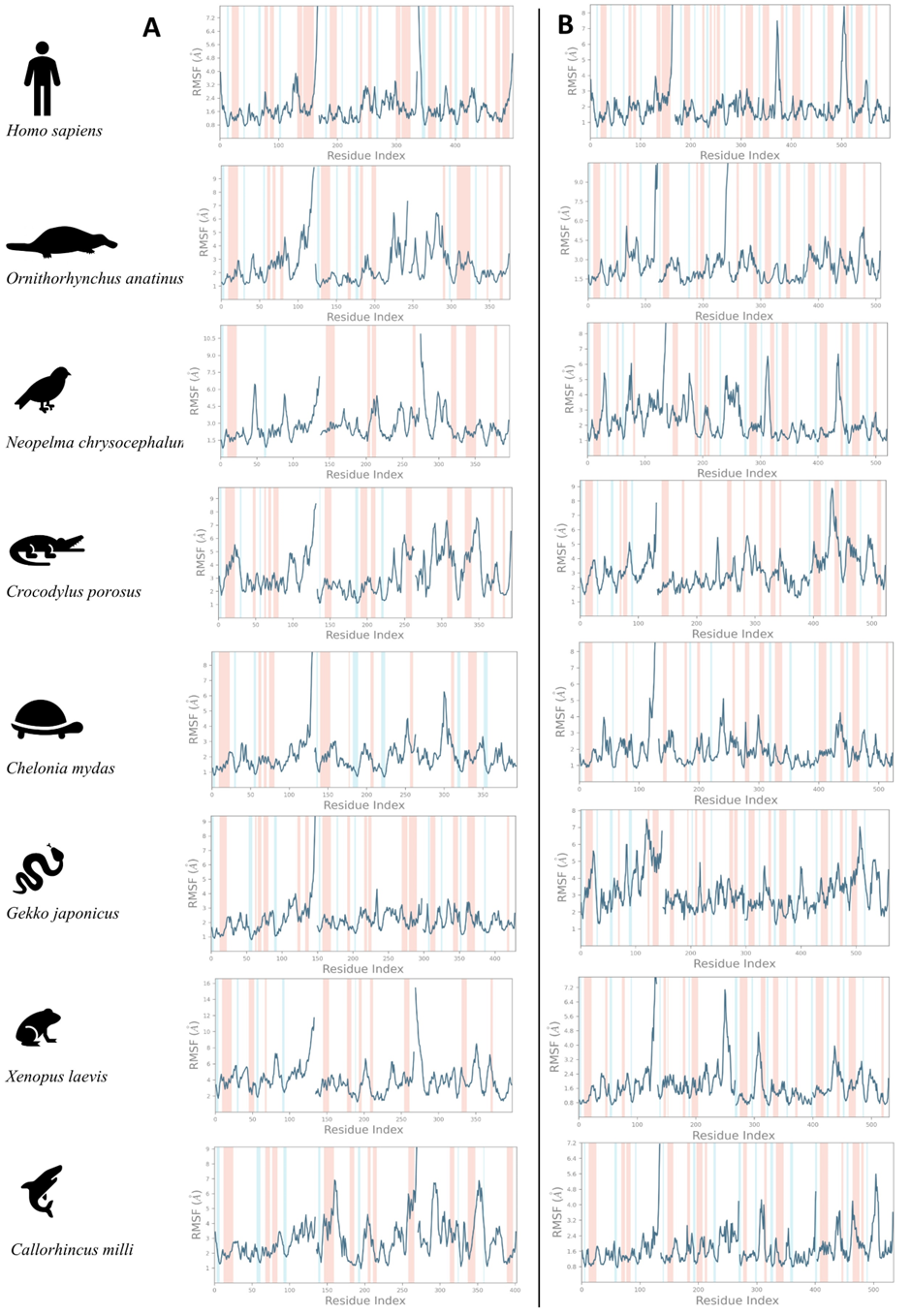
Figure S7

# Figure S7: The root mean square fluctuations (RMSF) of the trimeric and tetrameric protein across the trajectory

# Figure S8

**Figure S8:** The centrality measures highlighting the persistence nature of important residues across organisms for trimeric complexes

# Figure S9

**Figure S9:** The centrality measures highlighting the persistence nature of important residues across organisms for tetrameric complexes

#
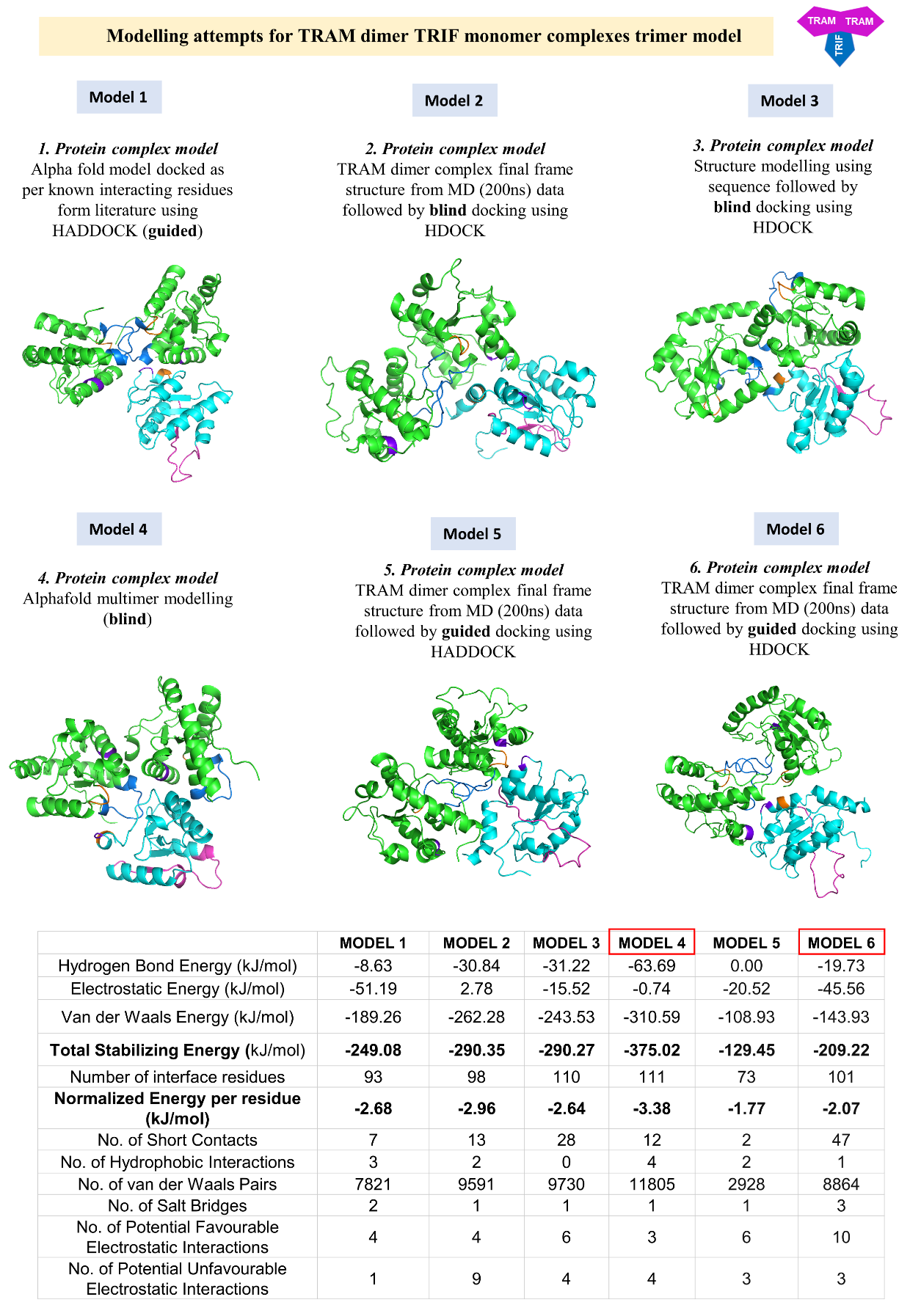
Figure S10

**Figure S10:** The structure of the various trimeric complex models and its energies

# Figure S11


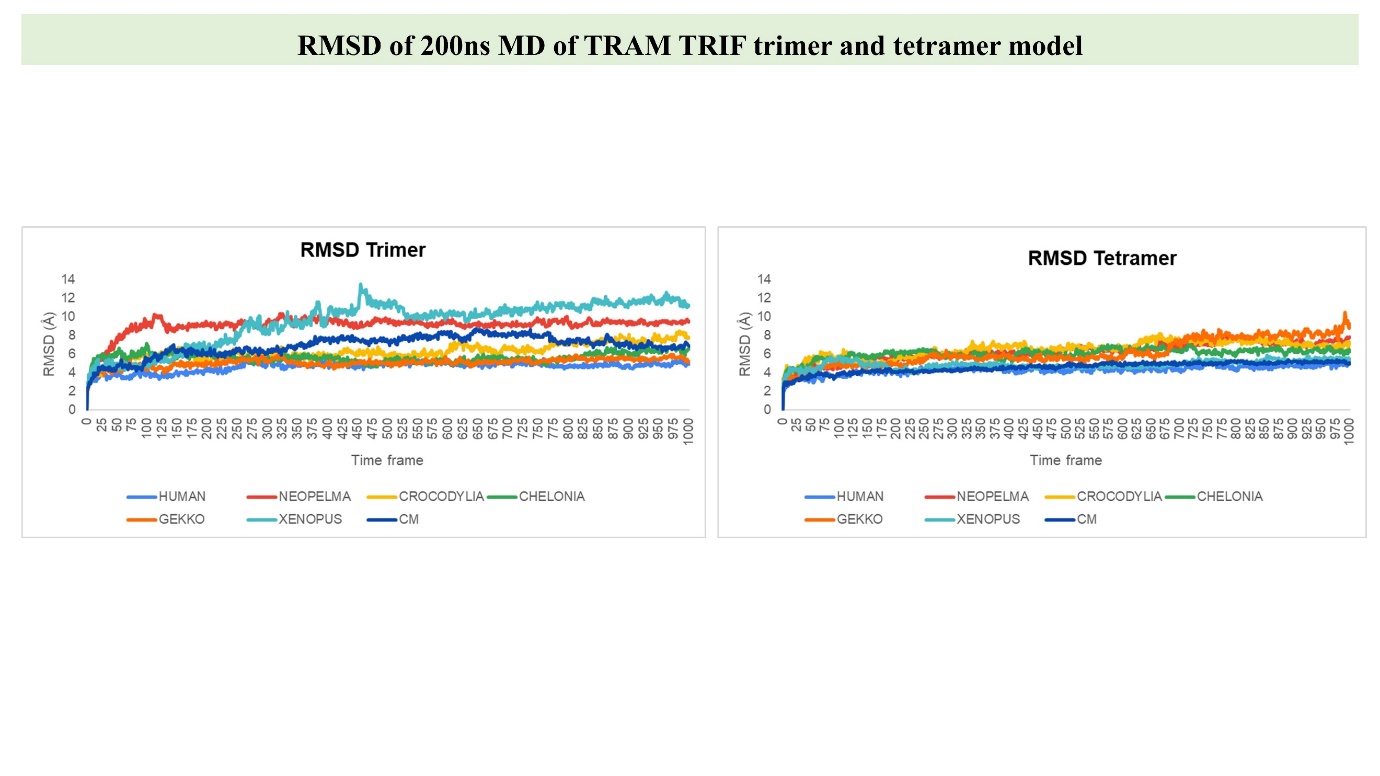


**Figure S11:** RMSD of 200ns molecular dynamics trajectory of TRAM and TRIF trimer and tetramer model. The 200ns plot has been converted to 1000 frames where each frame corresponds to 0.2 ns.
